# The Wave2 scaffold Hem-1 is required for transition of fetal liver hematopoiesis to bone marrow

**DOI:** 10.1038/s41467-018-04716-5

**Published:** 2018-06-18

**Authors:** Lijian Shao, Jianhui Chang, Wei Feng, Xiaoyan Wang, Elizabeth A. Williamson, Ying Li, Amir Schajnovitz, David Scadden, Luke J. Mortensen, Charles P. Lin, Linheng Li, Ariel Paulson, James Downing, Daohong Zhou, Robert A. Hromas

**Affiliations:** 1 Department of Pharmacology, University of Illinois Chicago, Chicago, IL 60612 USA; 2 Department of Pharmaceutical Sciences and Winthrop P. Rockefeller Cancer Institute, University of Arkansas for Medical Sciences, Little Rock, AR 72205 USA; 3 Department of Medicine and Pathology, University of Florida, Gainesville, FL 32610 USA; 4 Stem Cell and Regenerative Biology Department, Harvard University, Cambridge, 02138 MA USA; 5 Center for Regenerative Medicine, Massachusetts General Hospital, Boston, 02114 MA USA; 6 Harvard Stem Cell Institute, Cambridge, MA 02138 USA; 7 Regenerative Medicine Center, University of Georgia, Athens, GA 30602 USA; 8 Wellman Center for Photomedicine, Massachusetts General Hospital, Harvard Medical School, Boston, MA 02114 USA; 9 Department of Pathology and Laboratory, Medicine University of Kansas, Kansas City, 66160 KA USA; 10 Stowers Institute for Medical Research, Kansas City, MO 66160 USA; 11 Department of Pathology and Laboratory Medicine, St. Jude Children’s Research Hospital, Memphis, TN 38105 USA; 12 Department of Pharmacodynamics, University of Florida, Gainesville, FL 32610 USA; 13 Office of the Dean and the Cancer Center, Long School of Medicine, University of Texas Health Science Center, San Antonio, TX 78229 USA

## Abstract

The transition of hematopoiesis from the fetal liver (FL) to the bone marrow (BM) is incompletely characterized. We demonstrate that the Wiskott–Aldrich syndrome verprolin-homologous protein (WAVE) complex 2 is required for this transition, as complex degradation via deletion of its scaffold *Hem-1* causes the premature exhaustion of neonatal BM hematopoietic stem cells (HSCs). This exhaustion of BM HSC is due to the failure of BM engraftment of *Hem-1*^−/−^ FL HSCs, causing early death. The *Hem-1*^−/−^ FL HSC engraftment defect is not due to the lack of the canonical function of the WAVE2 complex, the regulation of actin polymerization, because FL HSCs from *Hem-1*^−/−^ mice exhibit no defects in chemotaxis, BM homing, or adhesion. Rather, the failure of *Hem-1*^−/−^ FL HSC engraftment in the marrow is due to the loss of c-Abl survival signaling from degradation of the WAVE2 complex. However, c-Abl activity is dispensable for the engraftment of adult BM HSCs into the BM. These findings reveal a novel function of the WAVE2 complex and define a mechanism for FL HSC fitness in the embryonic BM niche.

## Abstract (web summary)

Hematopoietic stem cells (HSCs) migrate from the fetal liver to the bone marrow (BM) during embryogenesis. Here the authors show that the WAVE2 complex scaffold Hem1 is required for engraftment of HSCs in BM, not through its canonical role regulating actin polymerization, but through c-Abl survival signaling.

## Introduction

Hematopoietic stem cells (HSCs) migrate from their sites of origin to the fetal liver (FL) on embryonic day (E) 9.5–10.5 in murine development^1–3^. This transient residence in the FL is essential for the maturation of adult HSCs and functional adult hematopoiesis^3–5^. After expansion in the FL, HSCs complete their epic journey by migrating to the bone marrow (BM) on E16.5 to 17.5^1–4^. Several key BM environmental signals mediate the migration of FL HSCs, including CXCL12, VEGF, Slit, SCF, collagen, N-cadherin, VCAM, selectins, and fibronectin^1,5–11^. However, the FL HSC intracellular signaling that drives the transition to BM hematopoiesis is poorly defined^1–3^. The two Wiskott–Aldrich syndrome verprolin-homologous protein complexes (WAVE1 and 2) are crucial regulators of cell movement, but their role in FL HSC migration has not been explored^12–14^.

The two heteropentameric WAVE complexes both can activate the actin-nucleating Arp2/3 complex in an intracellular location-specific manner^14–16^. The WAVE2 complex is composed of the ABI-1, SRA-1, BRK-1, HEM-2 (also termed NCKAP1), and WAVE2 proteins^14–16^. WAVE2 is the only WAVE complex expressed in HSCs^17^. The WAVE complexes are further distinguished by tissue-specific components (HEM-1 versus HEM-2)^18–20^ that are the inner membrane scaffolds upon which the WAVE complexes directionally assemble. In all hematopoietic cells, HEM-1 (also termed NCKAPL1) replaces HEM-2^18–21^. P-element insertion in *Drosophila Hem-2* resulted in failure of maternal RNA segregation, a cytoskeletal function, and subsequent embryo malformation and death^18^. Repression of HEM-1 in human neutrophils impairs, but does not completely abrogate their attractant-induced actin polymerization, polarity, and chemotaxis^20,21^. Moreover, a chemically induced point mutation in *Hem-1* in mice caused defective actin polymerization and defects in leukocyte development and function^21^. In addition to regulating the localization of the WAVE complexes, HEM-1 and HEM-2 regulate WAVE stability. When HEM-1 or HEM-2 is depleted in multiple model organisms, the other WAVE complex components are also degraded^21–24^. This co-dependent stability may be an important mechanism to prevent aberrant actin polymerization^21,22,24^. As well as actin polymerization and cell migration, the WAVE2 complex component ABI-1 propagates c-ABL signaling^25–30^. The SH3 domain of ABI-1 interacts with the proline-rich region of c-ABL and mediates the dimerization of c-ABL, which can activate c-ABL kinase activity^26,27^. c-ABL also feeds back to enhance WAVE complex activation^12,13,20,29^.

We examined the role of the WAVE2 complex scaffold *Hem-1* in the migration of FL HSC to the BM. Deletion of *Hem-1* resulted in degradation of the WAVE2 complex^21–24^, but surprisingly the migration of FL HSC to the fetal BM was not altered. Rather, after arriving in the fetal marrow niche, *Hem-1*^−/−^ FL HSC underwent apoptosis. Within 6–8 weeks *Hem-1*^−/−^ mice underwent marrow fibrosis and hematopoietic failure, and subsequently died. Neither FL nor young marrow *Hem-1*^−/−^ HSC could engraft irradiated wild-type (wt) adult mice. Without the WAVE2 complex present, Abi-1 was not present to activate downstream c-Abl signaling. Reconstituting c-Abl expression rescued *Hem-1*^−/−^ HSC survival in the adult marrow niche. This study implies that FL HSCs are not fit for BM occupancy until after a niche survival signal mediated by the WAVE complex through c-Abl. This defines a novel WAVE complex function, survival signaling, and sheds light on the regulation of the transition of hematopoiesis from the FL to the marrow during development.

## Results

### Premature death and hematopoietic defects in *Hem-1*^−/−^ mice

**Fig. 1:**
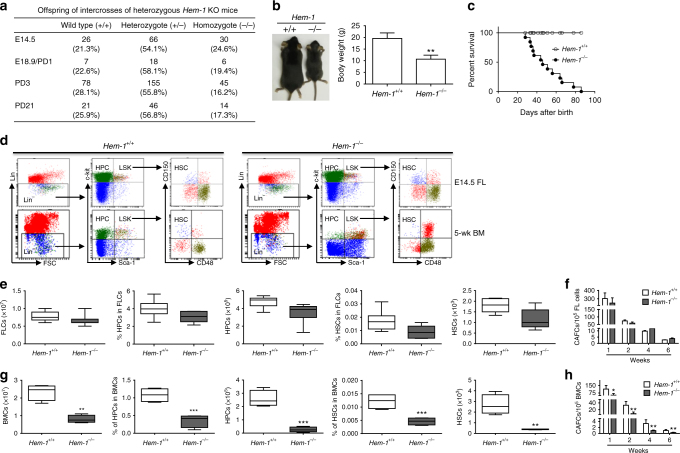
*Hem-1*^−/−^ mice exhibit decreased HPCs and HSCs, and die prematurely. **a** Genetic characterization of *Hem-1*^*+/*−^ offspring, demonstrating that *Hem-1*^−/−^ mice are born with appropriate Mendelian frequency. **b** *Hem-1*^−/−^ mice have growth retardation compared to littermate *Hem-1*^*+/+*^ mice (*Hem-1*^*+/+*^ mice, *n* = 6; *Hem-1*^−/−^ mice, *n* = 8, **p < 0.01, Student’s *t* test). **c** *Hem-1*^−/−^ mice die an average of 6 weeks after birth (*n* = 13, *p* < 0.001, log-rank test). **d** Flow cytometric analytic schematic of hematopoietic stem and progenitor cells in *Hem-1*^−/−^ and littermate *Hem-1*^*+/+*^ mice. (FSC: forward scattered light, Lin^−^: CD3e^−^/CD11b^−^/CD45R^−^/B220^−^/Ter-119^−^/Gr-1^−^, LSK: Lin^−^/Sca-1^+^/c-Kit^+^, HPC: Lin^−^/Sca-1^−^/c-Kit^+^, HSC: LSK/CD150^+^/CD48^−^). **e** E14.5 fetal liver hematopoietic stem and progenitor cells subsets are not different between *Hem-1*^−/−^ and littermate *Hem-1*^*+/+*^ mice (*n* = 10). **f** E14.5 FL cobblestone area-forming cells (CAFCs) are not different between *Hem-1*^−/−^ and *Hem-1*^*+/+*^ mice (*n* = 3). **g** Five-week *Hem-1*^−/−^ BM exhibits decreased hematopoietic stem and progenitor cell subsets (*n* = 5, **p < 0.01, ***p < 0.001, Student’s *t* test). **h** Five-week *Hem-1*^−/−^ BM CAFCs are reduced compared to littermate *Hem-1*^*+/+*^ mice (*n* = 3, *p < 0.05, **p < 0.01, Poisson statistics). Error bars represent the mean ± SD

We hypothesized that the hematopoietic-specific WAVE complex scaffold *Hem-1* is important for FL HSC transition to the BM. In the present study, *Hem-1* was constitutively deleted in a murine model to assess fetal HSC development and migration (Supplementary Fig. 1a–d). Constitutive deletion permitted study of whether Hem-1 was essential for the development of any other organ system outside the hematopoietic system. In addition, it ensured that all HSCs had the gene deleted, and therefore a small number of HSC escaping conditional deletion could not skew the study. Intercrosses of *Hem-1*^*+/*−^ mice produced *Hem-1*^*+/+*^, *Hem-1*^*+/*−^, and *Hem-1*^−/−^ E14.5 fetuses at the expected Mendelian ratio (Fig. 1a). However, *Hem-1*^−/−^ mice exhibited growth retardation and died prematurely after birth, with an average life expectancy of 6 weeks (Fig. 1b, c). These abnormalities were associated with a dramatic defect in BM hematopoiesis, including a significant reduction in the number of total BM nucleated cells (BMCs), BM phenotypic HSCs and hematopoietic progenitor cells (HPCs), and BM cobblestone area-forming cells (CAFCs) in 5-week-old *Hem-1*^−/−^ compared with littermate *Hem-1*^*+/+*^ mice of the same age (Fig. 1d–h). In addition, *Hem-1*^−/−^ mice developed a myelofibrosis-like disease with BM fibrosis, as demonstrated by an increase in reticulin staining; extra-medullary hematopoiesis, neutrophilia, and lymphopenia (Supplementary Fig. 2a–d). Heterozygote *Hem-1*^*+/*−^ mice developed normally, similar to *Hem-1*^*+/+*^ mice, and showed none of the abnormalities observed in *Hem-1*^−/−^ mice.

### *Hem-1*^*−/−*^ FL HSCs are unable to engraft BM

**Fig. 2:**
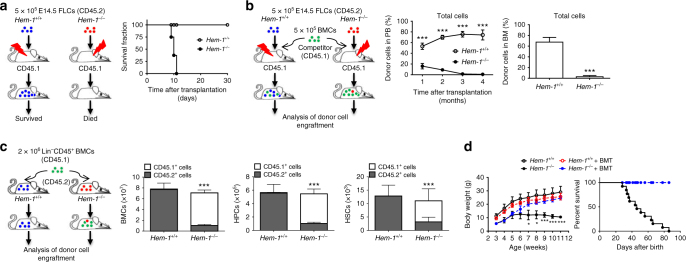
*Hem-1* deletion leads to an intrinsic functional defect in HSCs. **a** Schematic of rescue FLC transplantation where *Hem-1*^−/−^ CD45.2 HSCs failed to rescue lethally irradiated wild-type CD45.1 recipient mice (*n* = 8, *p* < 0.001, log-rank test). **b** E14.5 *Hem-1*^−/−^ CD45.2 FL HSCs could not compete with recipient-derived CD45.1 BM cells (BMC) in repopulating lethally irradiated *Hem-1*^*+/+*^ adult recipient mice. Blood was analyzed monthly after transplantation and marrow at 4 months post transplantation (*n* = 5, ***p < 0.001, Student’s *t* test). **c** Schematic of the competitive repopulation assay where exogenous littermate *Hem-1*^*+/+*^ CD45.1 HSCs efficiently rehabilitated the hematopoietic system in *Hem-1*^−/−^ CD45.2 mice via a reverse stem cell transplantation assay in non-ablated mice. BMCs, HPCs, and HSCs were analyzed at 4 months post transplantation (*n* = 8, ***p < 0.001, Student's *t* test). **d** Littermate *Hem-1*^*+/+*^ BM HSC rescued growth retardation and premature death when transplanted into non-ablated *Hem-1*^−/−^ mice (*n* = 12, *p < 0.05, ***p < 0.001, body weight: two-way ANOVA; percentage survival: log-rank test)

To investigate whether *Hem-1*^−/−^ mice die from premature exhaustion of BM HSCs as a result of an intrinsic defect in HSCs or a defect in their BM microenvironment, we performed rescue stem cell transplantation (SCT) by infusing E14.5 FLCs from *Hem-1*^*+/+*^ or *Hem-1*^−/−^ fetuses into lethally irradiated normal C57BL/6 congenic (CD45.1) mice. Transplantation of littermate *Hem-1*^*+/+*^ FL cells (FLCs) fully rescued the irradiated recipients, whereas all the recipients that received *Hem-1*^−/−^ FLCs died within 11 days after BMT (Fig. 2a). Competitive repopulation assays (CRAs) found that E14.5 FLCs from *Hem-1*^−/−^ mice failed to compete with normal CD45.1 BMCs for engraftment after SCT (Fig. 2b; Supplementary Fig. 3a, b). More importantly, we found that transplantation of *Hem-1*^*+/+*^ CD45.1 BMCs into non-ablated CD45.2 *Hem-1*^−/−^ mice at 3 weeks of age out-competed endogenous HSCs to repopulate their hematopoietic system, and rescued the *Hem-1*^−/−^ development defects and survival (Fig. 2c, d; Supplementary Fig. 3c, d). These results suggest that the lack of *Hem-1* does not affect fetal development, but causes growth retardation and premature death after birth due to an intrinsic defect in HSCs. The *Hem-1*^−/−^ BM HSCs exhibit premature exhaustion, which we postulated may be due to an inability of *Hem-1*^−/−^ FL HSCs to engraft in the BM.

### *Hem-1*^*−/−*^ FL HSCs can migrate to the BM

**Fig. 3:**
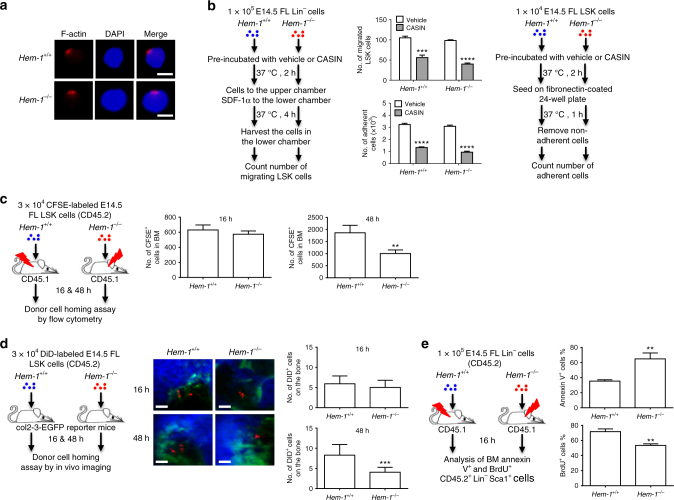
*Hem-1*^−/−^ hematopoietic stem and progenitor cells migrate and adhere normally. **a** Actin polymerization and capping are normal in E14.5 *Hem-1*^−/−^ FL LSK cells in response to SDF-1α stimulation. Representative microscopic images are shown (scale bar = 10 μm). **b** Migration towards SDF-1α and adherence to fibronectin were not different in E14.5 *Hem-1*^−/−^ FL Lin^−^ hematopoietic cells compared to *Hem-1*^*+/+*^ FL Lin^−^ cells, but they could be suppressed by inhibition of CDC42 with CASIN, a specific CDC42 inhibitor (*n* = 3, ***p < 0.001, ****p < 0.001, two-way ANOVA). **c** Homing of CFSE-labeled E14.5 *Hem-1*^−/−^ FL LSK hematopoietic cells to radiation-ablated adult marrow as measured by flow cytometry was not different from littermate E14.5 *Hem-1*^*+/+*^ FL LSK cells at 16 h after injection. However, there were decreased CSFE-labeled E14.5 *Hem-1*^−/−^ FL LSK cells present in the marrow at 48 h after injection (*n* = 5, **p < 0.01, Student’s *t* test). **d** Homing of DiD-labeled E14.5 *Hem-1*^−/−^ FL LSK cells to the osteoblastic niche of col2-3-EGFO reporter mice was normal compared to littermate *Hem-1*^*+/+*^ equivalent cells. However, after 48 h, there were decreased CSFE-labeled E14.5 *Hem-1*^−/−^ FL LSK cells present in the osteoblastic niche (*n* = 266–418 cells in three distinct mice, scale bar = 50 μm, ***p < 0.001, Student’s *t* test). **e** There were fewer E14.5 *Hem-1*^−/−^ FL Lin^−^ hematopoietic cells cycling as measured by BrDU incorporation and more undergoing apoptosis as measured by Annexin V expression after transplantation and migration to a radiation-ablated adult marrow than comparable littermate *Hem-1*^*+/+*^ cells (*n* = 5, **p < 0.01, Student’s *t* test)

FL HSCs transition to the BM starting around E16.5–17.5, and continues briefly after birth^1–3^. This transition requires significant cell migration and adherence. Therefore, we next examined whether *Hem-1* deletion leads to defects in FL HSC actin polymerization, migration, adherence, and homing to the BM. Unexpectedly, HSC-enriched Lin^−^/Sca-1^+^/Kit^+^ (LSK) E14.5 *Hem-1*^−/−^ FLCs showed no defects in F-actin polymerization, actin capping, and migration in response to the HSC chemokine stromal derived factor-1 alpha (SDF-1α), compared to littermate *Hem-1*^*+/+*^ equivalent cells (Fig. 3a, b). *Hem-1*^−/−^ FL Lin^−^ cells could adhere to fibronectin equally as well as the cells from *Hem-1*^*+/+*^ littermates (Fig. 3b**)**. In addition, E14.5 *Hem-1*^−/−^ FL LSK cells expressed levels of HSC adhesion and BM homing components (CXCR4, VLA-4, VLA-5, Tie2) equal to or greater than E14.5 *Hem-1*^*+/+*^ FL LSK cells (Supplementary Fig. 4). In contrast, neutrophils from *Hem-1*^−/−^ mice are defective in fMLP-stimulated F-actin polymerization, actin capping and migration, and adhesion to fibronectin as the cells from *Hem-1* mutant mice reported previously (Supplementary Fig. 5)^21^. Furthermore, we found that inhibition of CDC42 with a specific inhibitor, CASIN, suppressed both E14.5 *Hem-1*^*+/+*^ and *Hem-1*^−/−^ FL LSK cell adhesion and migration in vitro (Fig. 3b). These findings suggest that unlike neutrophils and other hematopoietic cells in adult mice, FL HSCs can migrate and home to the BM independent of the WAVE complex, perhaps via the CDC42 Wiskott–Aldrich syndrome protein (WASP) pathway. This is consistent with the observation that HSCs from WASP-deficient mice had decreased BM homing capability in association with a defect in adhesion to collagen^31^.

We next assessed whether FL hematopoietic stem/progenitor cells (HSPCs) were able to migrate to the BM in vivo after transplantation. 5-(and 6-)-Carboxyfluorescein succinimidyl ester (CFSE)-labeled E14.5 *Hem-1*^−/−^ CD45.2 FL LSK cells homed to the adult BM as well as their normal littermate counterparts after they were transplanted into lethally irradiated CD45.1 mice (Fig. 3c)^32^. There was no difference in the numbers of *Hem-1*^−/−^ E14.5 FL LSK cells in the BM 16 h after injection as equivalent *Hem-1*^*+/+*^cells. However, 48 h after arrival in the BM, *Hem-1*^−/−^ E14.5 FL LSK cells were half the numbers of their *Hem-1*^*+/+*^ counterparts (Fig. 3c). Next, we assessed *Hem-1*^−/−^ E14.5 FL LSK migration to the BM osteoblast niche^32^. There were equivalent numbers of 1,1′-dioctadecyl-3,3,3′,3′-tetramethylindodicarbocyanine perchlorate (DiD)-labeled *Hem-1*^−/−^ E14.5 CD45.2 FL LSK within the osteoblastic niche 16 h after injection into non-ablated CD45.1 col2-3-EGFP reporter mice as littermate *Hem-1*^*+/+*^ equivalent cells (Fig. 3d). However, 48 h after injection, there were more than twice the numbers of *Hem1*^*+/+*^ E14.5 FL LSK within the niche compared to equivalent *Hem-1*^−/−^ FL LSK cells (Fig. 3d)^32^, implying that the *Hem-1*^−/−^ FL LSK cells failed to survive and/or failed to proliferate once arriving in the BM niche. Using three-dimensional analysis, the distance to the nearest col2-3-EGFP osteoblast and endosteum was also not significantly different between *Hem-1*^*+/+*^ and *Hem-1*^−/−^ E14.5 FL LSK 16 and 48 h after the injection (Supplementary Fig. 6), indicating that *Hem-1*^−/−^ E14.5 FL LSK can adhere to the osteoblastic niche equally well as *Hem-1*^*+/+*^ E14.5 FL LSK. Interestingly, both cell types moved closer to the endosteum between the 16 and 48 h time points.

### *Hem-1*^*−/−*^ FL HSCs cannot survive in the BM

We then measured the ability of *Hem-1*^−/−^ E14.5 CD45.2 FL LSK cells to survive and proliferate after migration to the BM compared to littermate equivalent *Hem-1*^*+/+*^ cells (Fig. 3e). We found that *Hem-1*^−/−^ E14.5 CD45.2 FL LSK had both a higher rate of apoptosis and a decreased rate of cycling cells compared to equivalent cells from *Hem-1*^*+/+*^ littermate controls. This suggests that HSC-enriched LSK cells from the E14.5 *Hem-1*^−/−^ FL are just as capable of migrating to the BM and homing to the osteoblast niche as their normal counterparts, but cannot survive and proliferate once there^31^. Thus, *Hem-1* deletion does not impair FL to BM hematopoietic cell homing or adherence to the niche, suggesting that the WAVE2 complex has a distinct function in FL HSPCs besides regulating cell migration and adhesion by mediating survival and expansion after migration from the FL to the BM. This is consistent with the observation that knockdown of WAVE2 had no significant effect on HSC migration to the BM but prevented HSCs from expanding in the BM^17^.

**Fig. 4:**
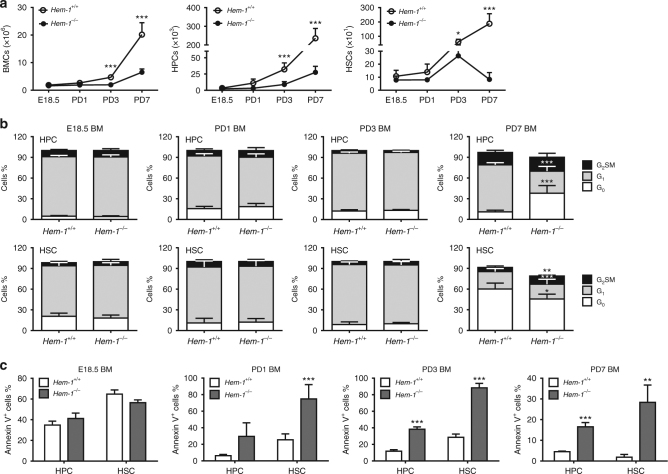
*Hem-*1 deletion results in a progressive decrease of HSCs mediated by increased apoptosis. **a** *Hem-1* deletion leads to the loss of HPCs and HSCs in a developmentally dependent manner. BM cells were harvested at E18.5, postnatal day (PD) 1, 3, and 7, and total BM cells (BMCs), HPCs, and HSCs were determined (*n* = 5, *p < 0.05, ***p < 0.001, one-way ANOVA). **b** HPCs and HSCs from *Hem-1*^−/−^ mice showed no significant difference in cell cycle distribution except PD7 compared to the cells from littermate controls (*n* = 5, **p < 0.01, ***p < 0.001, *χ*^2^ test). **c** *Hem-1*^−/−^ HPCs and HSCs had progressive increases in the fraction of apoptosis as measured by Annexin V staining compared to littermate controls (*n* = 5, **p < 0.01, ***p < 0.001, Student's *t* test)

However, the mechanism by which the WAVE complex regulates HSC expansion in the BM was unknown, and thus was studied further. First, we performed a detailed analysis of HSCs in the BM from E18.5 through postnatal day (PD) 1, PD3 and PD7 in both *Hem-1*^−/−^ and *Hem-1*^*+/+*^ fetuses and neonates. As expected, we found that the number of HSCs in the BM was low in E18.5 *Hem-1*^*+/+*^ fetuses and then increased rapidly from PD1 to PD7 in *Hem-1*^*+/+*^ neonates (Fig. 4a). By contrast, *Hem-1*^−/−^ neonates exhibited a moderate increase in the number of BM HSCs on PD3 and then an abrupt decrease on PD7 to a level similar to that in E18.5 fetuses (Fig. 4a). We then measured the cell cycle distribution and apoptotic fraction of *Hem-1*^−/−^ BM HSPCs compared with those of *Hem-1*^+/+^ littermate equivalent cells on E18.5, PD1, PD3 and PD7 using Ki-67/7-aminoactinomycin-D (7-AAD) and Annexin V staining, respectively. The cell cycle distribution was not significantly different between the cells from *Hem-1*^+/+^ and *Hem-1*^−/−^ littermates until PD7 (Fig. 4b). Interestingly, a high fraction of BM HSCs from both E18.5 *Hem-1*^−/−^ and *Hem-1*^+/+^ fetuses were apoptotic (Fig. 4c), indicating that FL HSCs arriving at the BM were not fit for their new environment. However, in *Hem-1*^+/+^ BM, the apoptotic fraction fell rapidly over the ensuing days, whereas in *Hem-1*^−/−^ BM the apoptotic fraction remained at a high level. This implies that there is an intrinsic survival signal the incoming FL HSCs require, but lack when *Hem-1* is deleted.

### Hem-1^**−/**−^ FL HSCs lack c-Abl survival signaling

We therefore sought to identify the FL HSC intrinsic survival signal required for engraftment after HSC transition to the BM. In addition to regulating actin polymerization, which mediates cell migration and adherence, the WAVE complex can also promulgate c-Abl signaling via c-Abl’s interaction with Abi-1^25–30^. Deletion of c-Abl results in defects in embryonic hematopoiesis resembling those seen here^33,34^, and constitutive activation of c-Abl via the t(9;22) increases HSC survival and proliferation and generates chronic myeloid leukemia (CML)^35–37^. Therefore, we investigated whether the defect in the *Hem-1*^−/−^ FL HSC transitioning to BM hematopoiesis was due to a lack of c-Abl survival signaling.

**Fig. 5:**
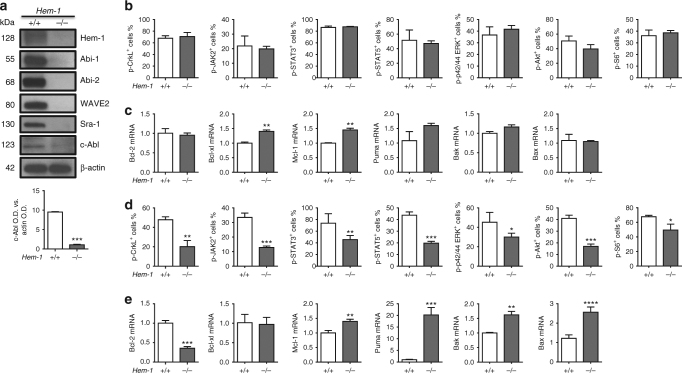
A defect in c-Abl signaling contributes to the impairment of *Hem-1*^−/−^ HSCs. **a** As expected, depletion of Hem-1 protein results in degradation of other WAVE2 components such as Abi-1, Abi-2, WAVE2, and Sra-1. Expression of c-Abl protein was also decreased in *Hem-1*^−/−^ E14.5 CD45^+^ Lin^−^ FL cells as measured by western blot. β-Actin was used as a loading control. Optical density (OD) averages of c-Abl signal normalized to actin (±S.D.) of three distinct western analyses (*n* = 3, ***p < 0.001, Student’s *t* test). **b** Flow cytometric analysis of the phosphorylation of downstream effectors of the c-Abl signaling pathway in E14.5 CD45^+^ Lin^−^ FL cells found no differences compared to littermate *Hem-1*^*+/+*^ cells (*n* = 9). **c** Quantitative RT-PCR expression analysis of regulators of apoptosis between *Hem-1*^*+/+*^ and littermate *Hem-1*^−/−^ E14.5 CD45^+^ Lin^−^ FL (*n* = 9, **p < 0.01, Student’s *t* test). **d** The phosphorylation of c-Abl downstream signaling pathway effectors in *Hem-1*^−/−^ PD3 Lin^−^ BM cells was reduced compared to littermate *Hem-1*^*+/+*^ equivalent cells (*n* = 9, *p < 0.05, **p < 0.01, ***p < 0.001, Student’s *t* test). **e**, Quantitative RT-PCR expression analysis of regulators of apoptosis found that pro-apoptotic effectors were increased and anti-apoptotic effectors were decreased in *Hem-1*^−/−^ PD3 Lin^−^ BM cells compared to littermate *Hem-1*^*+/+*^ equivalent cells (*n* = 9, **p < 0.01, ***p < 0.001, ****p < 0.0001, Student’s *t* test)

Without Hem-1 as the assembly scaffold, the other components of the WAVE2 complexes are reported to degrade^21,23,24^. As expected, deletion of the WAVE scaffold *Hem-1* here also resulted in degradation of the WAVE2 components Abi-1, Abi-2, WAVE2 and Sra-1 in FL Lin^−^ cells (Fig. 5a). As a result of the loss of its interacting partner Abi-1, the expression of c-Abl protein was significantly reduced in Lin^−^ cells from *Hem-1*^−/−^ FL Lin^−^ cells (Fig. 5a and Supplementary Fig. 7). Similar findings were also observed in PD3 BM Lin^−^ cells. In E14.5 *Hem-1*^−/−^ FL LSK cells, the loss of c-Abl did not affect the phosphorylation of Crkl, Jak2, Stat3, Stat5, Erk, Akt, and S6, downstream effectors of HSC survival and hematopoiesis (Fig. 5b, c and Supplementary Fig. 8)^35–37^. However, by PD3, *Hem-1*^−/−^ BM LSK cells exhibited a significant reduction in the phosphorylation of all of these signaling components (Fig. 5d and Supplementary Fig. 8). These results suggest that: (1) the c-Abl protein relies on the WAVE2 complex for stability in FL HSPCs^25–30^, and (2) there are other signals in FL HSPCs for survival besides c-Abl^38–40^. However, c-Abl signaling becomes essential at the transition of hematopoiesis from the FL to the neonatal BM. The differential phosphorylation status of downstream signalers in E14.5 *Hem-1*^−/−^ FL LSK cells and PD3 *Hem-1*^−/−^ BM LSK cells correlated with their expression of anti-apoptotic and pro-apoptotic proteins and the level of apoptosis (Fig. 5b–e and Supplementary Fig. 8). The PD3 *Hem-1*^−/−^ BM LSK cells had decreased Bcl-2 and increases in Puma, Bak, and Bax messages, indicating that there was significant apoptotic signaling in the *Hem-1*^−/−^ PD3 BM LSK cells compared to littermate *Hem-1*^*+/+*^ equivalent cells.

**Fig. 6:**
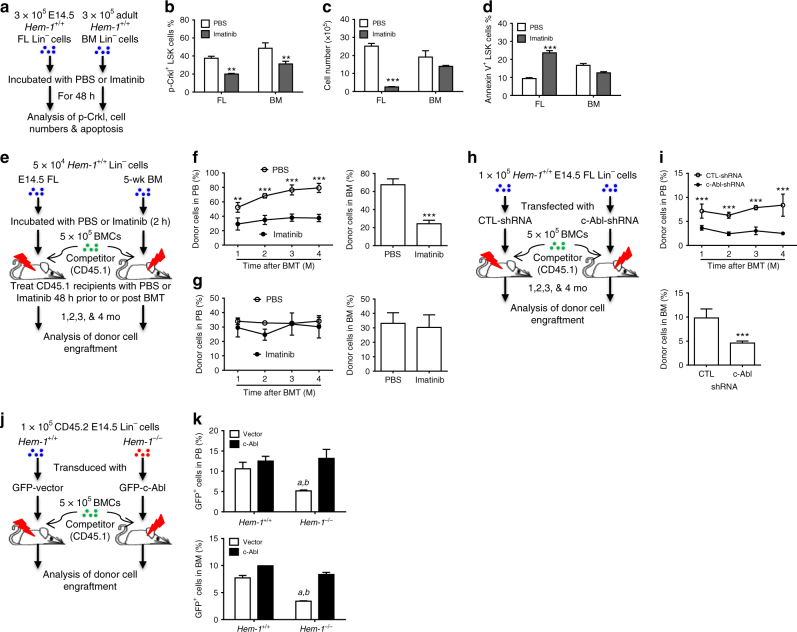
c-Abl is essential for engraftment of FL HSCs after homing to marrow. **a** Schematic of imatinib treatment of 5-week-old adult *Hem-1*^*+/+*^ BM and E14.5 FL Lin^−^ cells. **b** Imatinib treatment decreased phosphorylation of Crkl in both adult BM and FL Lin^−^ cells (*n* = 3, **p < 0.01, two-way ANOVA). **c** and (**d**) Imatinib treatment decreased cell growth and increased apoptosis only in the *Hem-1*^*+/+*^ E14.5 Lin^−^ FL cells but not adult *Hem-1*^*+/+*^ BM Lin^−^ cells (*n* = 3, ***p < 0.001, two-way ANOVA). **e** Schematic of Imatinib treatment of E14.5 *Hem-1*^*+/+*^ Lin^−^ FL cells or 5-week-old adult *Hem-1*^*+/+*^ Lin^−^ BM cells (CD45.2) competitively transplanted into radiation-ablated 5-week-old CD45.1 wild-type mice. The analysis of CD45.2 engraftment in the peripheral blood was performed monthly after transplantation. At 4 months post transplantation, CD45.2 BMCs were assessed to analyze the engraftment. **f** Imatinib treatment decreased the engraftment *Hem-1*^*+/+*^ E14.5 Lin^−^ FL cells in peripheral blood and bone marrow (*n* = 5, **p < 0.01, ***p < 0.001, Student’s *t* test). **g** Imatinib treatment has no effect on the engraftment ability of adult *Hem-1*^*+/+*^ Lin^−^ BM cells in peripheral blood and bone marrow (*n* = 5). **h** Schematic of lentivirally transduced shRNA against c-Abl (c-Abl-shRNA) or vector (CTL shRNA) in *Hem-1*^*+/+*^ E14.5 Lin^−^ CD45.2 FL cells competitively transplanted into radiation-ablated 5-week-old *Hem-1*^*+/+*^ CD45.1 mice. The analysis of CD45.2 engraftment in the peripheral blood was performed monthly after transplantation. At 4 months post transplantation, CD45.2 BMCs were assessed to analyze long-term engraftment. **i** Lentivirally transduced shRNA against c-Abl reduced engraftment of *Hem-1*^*+/+*^ E14.5 Lin^−^ FL cells into the blood and marrow of recipients (*n* = 5, ***p < 0.001, Student’s *t* test). **j** Schematic of transducing constitutively activated c-Abl (GFP-c-Abl or c-Abl) or vector (GFP-vector or Vector) into *Hem-1*^*+/+*^ and *Hem-1*^−/−^ E14.5 Lin^−^ cells in a competitive transplantation assay. **k** Over-expression of c-Abl rescued the defect in the engraftment ability in *Hem-1*^−/−^ Lin^−^ FL HSCs (*n* = 5, *a* *p* < 0.01 vs. *Hem-1*^*+/+*^ Vector, *b* *p* < 0.01 vs. *Hem-1*^−/−^ GFP-c-Abl, two-way ANOVA)

To determine whether the hematopoietic transition from the FL to the BM is dependent on c-Abl signaling for HSC survival, we incubated E14.5 FL and adult BM Lin^−^ cells from *Hem-1*^*+/+*^ mice with Imatinib (c-Abl kinase inhibitor)^35–37^, and then analyzed FL and BM LSK cell proliferation and apoptosis (Fig. 6a–d). Imatinib resulted in decreased cultured *Hem-1*^*+/+*^ FL LSK cell proliferation and increased apoptosis, but not in BM LSK cells. Significantly, the inhibition of c-Abl activity with Imatinib reduced the long-term engraftment of competitively transplanted *Hem-1*^*+/+*^ FL HSCs (Fig. 6e–i). The inhibition of c-Abl activity by imatinib had little effect on the engraftment of competitively transplanted adult *Hem-1*^*+/+*^ CD45.2 BM Lin^−^ cells, but markedly decreased the competitive engraftment of E14.5 *Hem-1*^*+/+*^ CD45.2 FL Lin^−^ cells (Fig. 6e–g; Supplementary Fig. 9a–d). To control the target specificity of imatinib, we next depleted endogenous c-Abl by transducing E14.5 *Hem-1*^*+/+*^ FL Lin^−^ cells with a lentiviral short hairpin RNA (shRNA) against c-Abl (Fig. 6h, i; Supplementary Fig. 9e, f). We demonstrated that depletion of c-Abl using a lentiviral shRNA in the *Hem-1*^*+/+*^ E14.5 CD45.2 FL Lin^−^ cells resulted in a marked decrease in their competitive engraftment in irradiated CD45.1 mice compared to equivalent cells transduced with a scrambled shRNA control. Finally, we examined whether the *Hem-1*^*−/*−^ HSC phenotype could be rescued by reconstitution of the expression of c-Abl. c-Abl was lentivirally expressed in *Hem-1*^−/−^ CD45.2 E14.5 FLCs, and these were tested for their ability to establish hematopoiesis in CRAs. The ectopic constitutive expression of c-Abl restored the ability of the *Hem-1*^−/−^ E14.5 CD45.2 FL Lin^−^ cells to competitively engraft lethally irradiated CD45.1 wt recipients, whereas lentiviral c-Abl had minimal effect on the engraftment of transplanted equivalent cells from littermate controls (Fig. 6j, k). These findings demonstrate that FL HSCs depend on c-Abl for survival and engraftment in the BM, and that *Hem-1*^−/−^ FL HSCs lack this signal.

## Discussion

In this study, we constitutively deleted *Hem-1* in mice to originally assess two questions: First, was Hem-1 function specific for hematopoietic cells, or were other organs affected by its deletion? Second, were Hem-1 and Hem-2 interchangeable, or did they have specific functions? In the initial characterization of the *Hem-1*^−/−^ mice we found that all other organ systems developed normally, and functioned into adulthood. The only defects were in the hematopoietic system. Thus, Hem-1 function was indeed specific for blood cells, and was not required for the normal development and function of any other organ systems. Second, consistent with the previous report by Park et al.^21^ that a *Hem-1* point mutation affected blood cell development, we found multiple hematopoietic defects in the *Hem-1*^−/−^ mice. Thus, Hem-2 cannot replace Hem-1 function in hematopoiesis.

The *Hem-1*^−/−^ FL HSC were essentially normal in numbers and proliferative function, but after migration to the marrow osteoblast niche, marrow HSC became rapidly depleted, resulting in a myelofibrosis-like phenotype, with anemia, myeloid metaplasia, and marrow reticulin. The *Hem-1*^−/−^ mice lacked other WAVE2 components, consistent with earlier findings in flies, amoeba, and mice, that WAVE complexes are obligate heteropentamers, probably to prevent unregulated actin polymerization, and cell paralysis^22–25^. Crystal structures of HEM-2 in the WAVE complex structure indicate that it likely attaches to the membrane and serves as a scaffold for the other WAVE components to assemble^16^. Thus, without the scaffold and the protection of the assembled heteropentamer, it is not surprising that the individual WAVE protein components are prone to destruction.

c-Abl-null mice also exhibit neonatal lethality, and if they survive they become runting, splenic, and marrow atrophy, with lymphopenia and increased susceptibility to infections, similar to the *Hem-1*^−/−^ mice here^33,34^. Consistent with previous reports, when Abi-1 is degraded in the *Hem-1*^−/−^ cells c-Abl fails to signal^25–29^, resulting in decreased phosphorylation of downstream c-Abl targets, such as Crkl, Stat3, and S6, both in FL and marrow stem/progenitor cells^35,36^. Depleting or inhibiting c-Abl inhibit wt FL HSC engraftment capability, but they do not harm engraftment of adult marrow HSC^35,36^. Restoring c-Abl expression to the FL *Hem-1*^−/−^ HSC rescues their marrow engraftment. Thus, there is a WAVE2/c-Abl survival signal required at the FL HSC to marrow transition that confers fitness to the HSC for marrow hematopoiesis, similar to thymic T cell selection.

Since the WAVE2 complex is not needed for FL HSC to migrate to the marrow osteoblast niche, there must be alternative pathways that can mediate appropriate actin polymerization for FL HSC migration and adherence. WASP complexes are a potential candidate to mediate an alternative pathway for actin polymerization besides WAVE utilized by the FL HSC to reach the marrow^38^. After activation by rac or cdc42, WASP also activates Arp2/3, and stimulates actin polymerization^39,40^. However, Rac1/2 and cdc42 deletion prevents FL HSC from reaching the marrow for transition of hematopoiesis, consistent with their importance in upstream regulation of actin polymerization, epistatically above both WAVE and WASP^39,40^. This is consistent with our data demonstrating that the cdc42 inhibition blocked FL HSC migration and adhesion.

The data here also have implications for the molecular pathogenesis of CML. The 9;22 chromosomal translocation in CML fuses BCR to c-Abl and results in HSC immortality, but these HSCs are addicted to c-Abl for survival^35,36^. This implies that the CML HSCs more closely resembles the FL HSCs than the marrow HSCs. It is therefore possible that WAVE2 might be important for BCR-Abl signaling, and WAVE2 could be an additional target for therapy in CML. In addition, this finding suggests that pregnant CML patients should not be treated with a c-Abl inhibitor at late gestation. It might inhibit the survival of fetal HSC after migration from the FL to the marrow, and result in a decreased HSC population into adulthood. Exposure to imatinib during pregnancy results in an increased incidence of fetal malformations^41^, but there is little data on blood counts as children of such pregnancies mature^41^. Such children could have a higher risk of marrow aplasia or myelofibrosis in adulthood.

In summary, the scaffold upon which the WAVE2 complex assembles in hematopoietic cells, Hem-1, is required for the engraftment of FL HSC in the marrow during embryonic development. Surprisingly, the engraftment failure of *Hem-1*^−/−^ FL HSC is not due to decreased actin polymerization resulting in poor migration or adherence to the marrow niche. Rather, this engraftment requires c-Abl signaling, which is lost when its partner Abi-1 is degraded with the rest of the WAVE complex when *Hem-1* is deleted. After the FL HSC has engrafted the marrow, the c-Abl survival signal is no longer needed, indicating that FL HSCs are specifically for and modified by the marrow microenvironment.

## Methods

### Model generation

For the generation of *Hem-1*-deleted mice, a mouse *Hem-1* genomic clone was obtained from the mouse 129s6/Sv BAC library^42^. A *lacZ-neo* cassette, in which the neomycin phosphotransferase gene is linked to the *lacZ* gene placed between the independent ribosomal entry sequences and an SV40 polyadenylation signal, replaced a sequence covering the *Hem-1* coding sequence of the first exon. The gene-targeting construct was electroporated into embryonic stem (ES) cells, and the cells were selected with neomycin. Recombinant ES cell clones were then injected into C57BL/6 mouse blastocysts, and *Hem-1*^*+/*−^ mice were obtained using standard protocols. All of the mice were backcrossed to C57BL/6J mice for more than eight generations before being used in our study. Constitutive deletion was chosen to: (1) ensure complete *Hem-1* deletion in embryonic HSC, and (2) analyze whether Hem-1 had any non-hematopoietic effects.

Male C57BL/6J (CD45.2) mice, B6.SJL-*Ptprc*^*a*^*Pep3*^*b*^/BoyJ (CD45.1) mice, and Col2.3-GFP mice were purchased from Jackson Laboratories (Bar Harbor, MA, USA). The mice were housed at the University of Arkansas for Medical Sciences (UAMS) and Harvard University AAALAC-certified animal facilities. The animals received food and water ad libitum. All mice were used at approximately 8–12 weeks of age. The Institutional Animal Care and Use Committees of UAMS and Harvard University approved all experimental procedures used in this study.

### Genotyping

Genomic DNA was isolated from mouse tails or embryo brains and digested in DNA lysis buffer (VIAGEN Biotech, Los Angeles, CA, USA)^42,43^. The genotype of each mouse or embryo was determined by PCR using *LacZ* and *Hem-1* primers. The *LacZ* forward (LACZFWD: 5′-TTCACTGGCCGTCGTTTTACAACGTCGTGA-3′) and *LacZ* reverse (LACZREV: 5′-ATGTGAGCGAGTAACAACCCGTCGGATTCT-3′) primers recognize the *LacZ* reporter gene from the knockout vector. The *Hem-1* forward (HEMFWD: 5′-GGTTGGGTGGGAAAGAGAATATTTGTGTTG-3′) and *Hem-1* reverse (HEMREV: 5′-TCACAGCCCCAAACTACCTTAGAAAACACC-3′) primers recognize the *Hem-1* gene. The *LacZ* product is 364 bp and the *Hem-1* product is 228 bp, as shown in Supplementary Fig. 1b.

### Peripheral blood counts and organ histology

Blood was obtained through retro-orbital bleeding and transferred to ethylenediaminetetraacetic acid (EDTA)-coated tubes^43–45^. Peripheral blood cell numbers were determined using a Vet Abc Hematological analyzer (Scil Animal Care, Gurnee, IL, USA). Tibiae, spleen, and lung from 5-week-old *Hem-1*^+/+^ or *Hem-1*^−/−^ mice were fixed in 4% paraformaldehyde (Fisher Scientific, Pittsburgh, PA, USA) for 24 h. Tibiae were decalcified in 14% EDTA for 7 days. The bones were then embedded in paraffin and 5-μm longitudinal sections were obtained. After de-paraffinization and rehydration, the sections were processed for staining with hematoxylin and eosin for the histologic assessment of organ morphology, silver for reticulin, or myeloperoxidase for myeloid cells.

### Isolation of BM and FL hematopoietic cell subsets

The femora and tibiae were harvested from mice immediately after they were euthanized with CO_2_^43–45^. BM cells were flushed from the bones into Hank's balanced salt solution (HBSS) containing 2% fetal calf serum (FCS) using a 21-gauge needle and syringe. FLCs were obtained from E14.5 embryos. The cells were centrifuged through Histopaque 1083 (Sigma, St. Louis, MO, USA) to isolate mononuclear cells (MNCs). For the isolation of Lin^−^ cells, MNCs from the BM or FL were incubated with biotin-conjugated rat antibodies specific for murine CD3e, CD11b, CD45R/B220, Ter-119, and Gr-1 (CD11b antibody was excluded in FL MNCs). The labeled mature lymphoid and myeloid cells were depleted twice by incubation with goat anti-rat IgG paramagnetic beads (Thermo Fisher Scientific, Waltham, MA, USA) at a bead:cell ratio of 4:1. Cells binding to the paramagnetic beads were removed with a magnetic field. The negatively isolated Lin^−^ cells were washed twice with 2% FCS/HBSS and re-suspended in complete medium (RPMI-1640 medium supplemented with 10% FCS, 2 mM l-glutamine, 10 µM HEPES buffer, and 100 U/ml penicillin and streptomycin) at 1 × 10^6^ cells/ml. LSK cells (Lin^−^/Sca-1^+^/c-kit^+^ cells) were sorted with an Aria II cell sorter (BD Biosciences, San Jose, CA, USA) after the Lin^−^ cells were pre-incubated with anti-CD16/32 antibody to block the Fcγ receptors and then stained with anti-Sca-1-PE and c-Kit-APC antibodies. Dead cells were excluded by gating out the cells stained with propidium iodide (PI).

### Analysis of the frequencies of hematopoietic cell subsets by flow cytometry

MNCs from BM or FL were pre-incubated with biotin-conjugated anti-CD3e, anti-CD45R/B220, anti-Gr-1, anti-CD11b, and anti-Ter-119 antibodies, with anti-CD16/32 antibody to block the Fcγ receptors (CD11b antibody was excluded in FL MNCs)^43–45^. The cells were then stained with streptavidin-fluorescein isothiocyanate (FITC) and anti-Sca-1-PE-Cy7, c-Kit-APC-Cy7, CD150-APC, and CD48-Pacific Blue antibodies. The frequencies of HPCs (Lin^−^/Sca-1^−^/c-kit^+^), LSK cells (Lin^−^/Sca-1^+^/c-kit^+^), and HSCs (CD150^+^/CD48^−^/LSK) were analyzed using an Aria II cell sorter. For each sample, approximately 5 × 10^5^ to 1 × 10^6^ cells were acquired and the data were analyzed using the BD FACSDiva 6.0 software (BD Biosciences) and the FlowJo software (FlowJo, Ashland, OR, USA) . The number of the different hematopoietic cell populations in each mouse was calculated by multiplying the total number of MNCs harvested from each mouse or embryo with the frequencies of each population of MNCs. The information for all antibodies used in the staining is provided in Supplementary Table 1.

### CAFC assay

Stromal cell feeder layers were prepared by seeding 10^3^/well FBMD-1 stromal cells in each well of flat-bottom 96-well plates (Falcon, Lincoln Park, NJ, USA)^44,45^. One week later, BMCs re-suspended in CAFC medium (Iscove’s modified Dulbecco's medium (MDM) supplemented with 20% horse serum, 10^−5^ M hydrocortisone, 10^−5^ M 2-mercaptoethanol, 100 U/ml penicillin, and 100 μg/ml streptomycin) were overlaid on these stromal layers in six threefold serial dilutions. Twenty wells were plated for each dilution to allow limiting dilution analysis of the precursor cells forming hematopoietic clones under the stromal layer. The cultures were fed weekly by changing one-half of the media. The frequencies of CAFCs were determined at weekly intervals. Wells were scored positive if at least one dark-phase hematopoietic clone (containing five or more cells) was observed. The frequency of CAFCs was then calculated using Poisson statistics as described previously^3,4^.

### Ionizing irradiation

Male CD45.1 mice at 8 to 10 weeks of age were exposed to a lethal dose (9.5 Gy) of total body irradiation in a J.L. Shepherd Model Mark I ^137^Cesium γ-irradiator (J.L. Shepherd, Glendale, CA, USA) at a dose rate of 1.080 Gy/min for BMT preconditioning. The mice were irradiated on a rotating platform.

### Timed mating and non-competitive SCT

Two *Hem-1*^+/−^ females in estrous were put together with one *Hem-1*^+/−^ male and checked for vaginal plugs the next morning, designated as 0.5 days post-coitum (dpc)^43–45^. At 14.5 dpc (E14.5), the FLs were dissected from the fetuses, and single-cell suspensions were prepared in HBSS. A total of 5 × 10^5^ FLCs pooled from three to four *Hem-1*^+/+^ or *Hem-1*^−/−^ embryos were transplanted into lethally irradiated CD45.1 recipient mice. The recipient mice were monitored up to 30 days after transplantation, and the number of dead/moribund mice was recorded on a daily basis. The genotypes were determined using a small amount of brain tissues from the fetuses prior to the transplant, prepared, and genotyped as described above.

### Competitive repopulation assay

A total of 5 × 10^5^ FLCs were pooled from three to four E14.5 Hem-1^+/+^ and Hem-1^−/−^ embryos^43–45^. The cells were mixed with 5 × 10^5^ competitive BMCs pooled from three CD45.1 mice and then transplanted into lethally irradiated CD45.1 recipients via retro-orbital injection of the venous sinus. Donor cell engraftment was determined at 1, 2, 3, and 4 months after transplantation by immunostaining of the cells in the recipient peripheral blood with FITC-conjugated anti-CD45.2; phycoeythrin (PE)-conjugated anti-B220, CD11b, and Gr-1; and APC-conjugated anti-B220 and CD3e antibodies and analyzed by flow cytometry. Four months after transplantation, the BMCs from the recipient mice were used to analyze the engraftment ability of the donor cells. Donor-derived HPCs and LSK cells from the BM were further evaluated using an Aria II flow cytometer. The information for all antibodies used in the staining is provided in Supplementary Table 1.

### Non-ablative SCT

Two million Lin^−^CD45^+^ cells were sorted from 6-week-old to 8-week-old CD45.1 mice and retro-orbitally injected into 3-week-old Hem-1^+/+^ and Hem-1^−/−^ mice^43–45^. The body weights of recipient Hem-1 mice were recorded weekly, and mouse survival was monitored daily. At 1, 2, 3, and 4 months after transplantation, the donor cell engraftment in the peripheral blood and BM was determined as described above.

### Homing assay

Thirty thousand FL LSK cells from E14.5 Hem-1^+/+^ and Hem-1^−/−^ embryos were stained with 5 μM CFSE-mixed isomers (Thermo Fisher Scientific, Waltham, MA, USA) for 10 min according to the manufacturer’s instructions^32,46^. The stained cells were re-suspended in 100 μl IMDM medium containing 30,000 cells and retro-orbitally transplanted into lethally irradiated male CD45.1 recipient mice. The mice were euthanized at 16 and 48 h after transplantation. The BMCs were harvested and analyzed by flow cytometry using an LSRII flow cytometer (BD Bioscience) for the presence of CFSE^+^ cells in PI-negative cells. The absolute numbers of CFSE^+^ cells in the BM from femora and tibiae were calculated by multiplying the total numbers of BMCs by the percentage of CFSE^+^ cells.

### Intra-vital microscopy

E14.5 FL LSK cell micro-localization in the BM, including the three-dimensional distance to the nearest col2-3-EGFP osteoblast and endosteum, was analyzed and measured by intra-vital microscopy using previously described equipment and procedures^6,32,46^. Thirty thousand LSK cells were labeled with DiD (Thermo Fisher Scientific, Waltham, MA, USA) and injected into non-irradiated Col2.3-GFP transgenic mice. A defined region of the calvarium BM cavity (4 × 6 mm^2^) was scanned using a confocal/two-photon hybrid microscope as previously described, allowing the visualization of DiD^+^ LSK cells and GFP^+^ cells. The cells were imaged at two time points post transplant, 16 and 48 h. To evaluate the distance between the transplanted FL LSK cells and the osteoblastic and endosteal surfaces, we computed values as described previously^6^. The shortest distance from a transplanted cell to a *Col2.3-GFP* osteoblast or the bone endosteal surface was determined in three dimensions using the Pythagorean theorem. Three recipient mice were analyzed per time point and donor genotype. DiD-labeled cells were identified and distinguished from auto-fluorescent cells using two confocal images at 633 nm (650–760 nm detection) and at 532 nm (560–640 nm detection). A 330 0.9NA water-immersion objective (Lomo) was used for all imaging. For three-dimensional analysis of the BM cavity, Z-stacks were acquired at 1–3 mm steps. A PCI-based image capture board (Snapper24, Active Silicon) was used to acquire up to three channels simultaneously using iPhoton32 software that was developed in-house running under Mac OS X.

### Apoptosis assay

BMCs were incubated with anti-CD16/32 at 4 °C for 15 min to block the Fc-γ receptors and then stained with antibodies against various cell-surface markers in the dark^44,45^. After Annexin V staining with a kit from BD Pharmingen (San Diego, CA, USA) according to the manufacturer’s instructions, the apoptotic cells within different hematopoietic cell populations were analyzed with an Aria II flow cytometer.

### Cell cycle assay

BMCs (1 × 10^6^) from E18.5, PD1, PD3, and PD7, mice were first stained with antibodies against various cell-surface markers and fixed and permeabilized using the Fixation/Permeabilization Solution from BD Biosciences (San Diego, CA, USA). The cells were subsequently stained with anti-Ki-67-FITC antibody and 7-AAD (Sigma) and then analyzed by flow cytometry^44,45^.

### BrdU incorporation assay

Lin^−^ cells (CD45.2) (1 × 10^5^) from E14.5 Hem-1^+/+^ and Hem-1^−/−^ embryos were transplanted into lethally irradiated recipient mice (CD45.1)^44,45^. Sixteen hours later, the recipient mice were euthanized, and 1 × 10^6^ Lin^−^ cells from the femora and tibiae were incubated with 5-bromo-2′-deoxyuridine (BrdU) (10 µM in 10% fetal bovine serum-DMEM medium) for 2.5 h. BrdU incorporation was measured by flow cytometry using the FITC BrdU Flow Kit from BD Biosciences (San Diego, CA, USA) according to the protocol provided by the manufacturer after the cells were stained with antibodies against various cell-surface markers. The BrdU^+^ cells within the Lin^−^ Sca-1^+^ cell population were expressed as percentages of CD45.2^+^ cells.

### Migration assays

In vitro migration in response to SDF-1α was analyzed as previously described^47^. Lin^−^ cells (1 × 10^5^) from FL of E14.5 Hem-1^+/+^ and Hem-1^−/−^ embryos were pre-incubated with vehicle or 10 µM CASIN (Sigma) for 2 h to inhibit the activity of CDC42. They were plated in the upper well of a 24-well Transwell chamber separated with a filter containing 5.0 μm pore size (Corning, Corning, NY, USA) in IMDM medium with 2% bovine serum albumin (BSA). After a 4-h incubation against an SDF-1 gradient (100 ng/ml) in the lower chamber, all cells that migrated through the filter were collected. These cells were then stained with c-Kit-APC and Sca-1-PE and analyzed using an LSRII flow cytometer (BD Bioscience) for the percentages of LSK cells within PI-negative cells. The numbers of migrating LSK cells were calculated by multiplying the total number of migrating Lin^−^ cells by the percentage of migrating LSK cells.

### Adhesion assays

To determine the ability of hematopoietic cells to adhere to a substrate in vitro, 10,000 FL LSK cells from E14.5 Hem-1^+/+^ and Hem-1^−/−^ embryos in Stemspan medium with 10 ng/ml thrombopoietin (TPO) and 10 ng/ml stem cell factor (SCF) were plated in triplicate on 24-well non-tissue culture-treated plates previously coated with fibronectin (CH-296, 20 μg/ml; Clontech Laboratories, Mountain View, CA, USA)^47^. The cells were incubated for 1 h at 37 °C, after which the supernatant was removed, and the wells were washed once with phosphate-buffered saline (PBS) to remove non-adherent cells. The number of adherent cells was counted under a light microscope.

### F-actin fluorescence staining

To characterize F-actin polymerization and capping, FL LSK cells from E14.5 Hem-1^+/+^ and Hem-1^−/−^ embryos were serum-starved in HBSS and stimulated with the chemokine SDF-1α (100 ng/ml) for 10 s^20^. The cells were fixed with 4% paraformaldehyde for 15 min and permeabilized with 0.1% Triton X-100 (Sigma) for 15 min. After blocking in 2% BSA, the cells were stained with tetramethylrhodamine-conjugated phalloidin (Thermo Fisher Scientific, Waltham, MA, USA) and 4',6-diamidino-2-phenylindole (DAPI) (Sigma) and mounted for fluorescence imaging analysis on a Zeiss fluorescence microscope equipped with a ×40 oil-immersion objective lens, a AxioCam MRm camera, and the AxioVision Rel. 4.8 software (Jena, Germany). The images shown are representative of more than 100 cells examined for each genotype.

### Neutrophil F-actin adhesion, migration, and polarization assays

Neutrophil isolation was performed as follows: BM cells (2 × 10^7^) from 5-week-old Hem-1^+/+^ and Hem-1^−/−^ mice were suspended in 3 ml HBSS-EDTA buffer and transferred on the top of pre-prepared Percoll gradient separation solution containing 78% Percoll (1.11 g/ml), 69% Percoll (1.09 g/ml), and 52% Percoll (1.083 g/ml)^20,47^. Cells were separated by centrifugation at 1500 x *g* for 30 min. The neutrophil layer was collected from the 78/68% Percoll interface and red blood cells were lysed with 0.83% NH_4_Cl solution. Neutrophils were counted and used for testing the capacities of adhesion, migration, and polarization.

For the F-actin polymerization and capping assay, neutrophils (2 × 10^5^) from 5-week-old Hem-1^+/+^ and Hem-1^−/−^ mice were serum-starved in PBS and stimulated with 10 nM fMLP (Sigma) for 120 s. The cells were then fixed with 4% paraformaldehyde for 15 min and permeabilized with 0.1% Triton X-100 (Sigma) for 15 min. After blocking in 2% BSA, the cells were stained with FITC-conjugated phalloidin (Thermofisher Scientific) and DAPI (Sigma), and mounted for fluorescence imaging analysis on a Zeiss fluorescence microscope equipped with a ×40 oil-immersion objective lens, a AxioCam MRm camera, and the AxioVision Rel. 4.8 software (Jena, Germany). The number of cells with F-actin capping was counted in 10 different fields under a ×40 oil-immersion objective lens. Percentages of cells with F-actin capping cells were presented. Images shown are representatives of more than 100 cells examined for each genotype.

For the neutrophil adhesion assay, neutrophils (2 × 10^5^) isolated from 5-week old Hem-1^+/+^ and Hem-1^−/−^ mice were plated in triplicate on 24-well non-tissue culture-treated plates previously coated with fibronectin (CH-296, 20 μg/mL; Clontech Laboratories). Cells were incubated for 2 h at 37 °C, after which the unattached cells were removed by washing with PBS. The numbers of adherent neutrophils were counted under light microscope.

For the neutrophil migration assay, neutrophils (1 × 10^5^) isolated from 5-week old Hem-1^+/+^ and Hem-1^−/−^ mice were plated in the upper well of a 24-well transwell chamber separated with a filter containing 5.0 μm pore size (Corning, Corning, NY, USA) in DMDM medium with 2% BSA. After 4-h incubation against an SDF-1α gradient (100 ng/ml) at the lower chamber, cells that migrated through the filter were collected and counted under light microscopy. In addition, neutrophil migration was also measured using a 96-well chamber with polycarbonate filters (Neuro Probe, Inc., Gaithersburg, MD, USA) in response to fMLP stimulation. Briefly, 29 μL fMLP (10 nM) was added to the bottom wells of the 96-well plate. Polycarbonate filters with a 3.0 μm pore size were placed between the lower plate and upper plate of the chamber. Fifty microliters of medium containing neutrophils (1 × 10^5^) were added to the top plate. The chamber was incubated for 1 h at 37 °C and 5% CO_2_. Non-migrating cells on the top of the filter were removed by gentle scraping. The number of migrated cells in the lower plate was counted under three different 200× fields and mean values were presented.

### Intracellular phospho-flow cytometric analysis

One million cells from E14.5 FLs or PD3 BMs were stained with various antibodies, fixed, and permeabilized using the Fixation/Permeabilization Solution from BD Pharmingen (San Diego, CA, USA)^44,45^. The cells were subsequently stained with anti-p-Crkl, p-JAK2, p-STAT3, p-STAT5, p-p42/44 ERK, p-Akt ser473, and p-S6 antibodies and then analyzed using a flow cytometer. The information for all antibodies used in the staining is provided in Supplementary Table 1.

### Imatinib administration

Imatinib (Selleckchem.com, Houston, TX, USA) was re-suspended in Dulbecco’s phosphate-buffered saline at 10 mmol/L. Lin^−^ cells (3 × 10^5^) from E14.5 FLs or 5-week-old BMs were incubated with 5 μmol/L imatinib or vehicle. Forty-eight hours later, p-Crkl, apoptosis, and the cell cycle were analyzed. For transplantation, 5 × 10^4^ Lin^−^ cells from E14.5 FLs or 5-week-old BM were incubated with 5.0 μmol/L imatinib or vehicle for 1 h at 37 °C. The treated cells along with 5 × 10^5^ recipient-derived BMCs were transplanted into lethally irradiated recipient mice that were treated with imatinib (100 mg/kg) for 48 h before and after transplantation. At 1, 2, 3, and 4 months after transplantation, donor cell engraftment in the peripheral blood and BM was determined as described above.

### Quantitative real-time PCR

Total cellular RNA was extracted from approximately 2000 sorted LSK cells using the Zymo Research Quick-RNA Micro Prep Kit (The Epigenetics Company, Irvine, CA, USA) according to the manufacturer’s instructions. First-strand cDNA was synthesized in a final volume of 20 μL using the Superscript III First-Strand Synthesis System (Thermo Fisher Scientific, Waltham, MA, USA)^44,45^. To measure Bcl-2, Bcl-xl, Mcl-1, Puma, Bak, Bax, Cxcr4, Vla-4, Vla-5, and Tie2 mRNA expression in LSK cells, quantitative real-time PCR (qRT-PCR) analyses were performed using a SYBR Green mix on an ABI StepOnePlus Real-Time PCR System (Applied Biosystems, Foster City, CA, USA). The 20-µL PCR reaction was prepared as follows: 10 µL of 2× SYBR Green PCR master mix, 0.4 µL of 10 µM of the appropriate forward and reverse primers, 7.6 µL RNase-free water, and 2 µL cDNA template. A negative control (no DNA template) was also performed for each master mix prepared. The qPCR was performed with 10 min at 95 °C, followed by 40 cycles of 15 s at 95 °C and 1 min at 60 °C. A dissociation melting curve was generated using temperatures from 60 °C to 95 °C. An arbitrary unit was calculated by the comparative CT method according to the CT values of the internal control. Mouse hypoxanthine guanine phosphoribosyl transferase was used as a constitutively expressed internal reference for mouse mRNA. The data are representative of two independent experiments performed in triplicate. The sequences for all the primers used in the qRT-PCR assays are shown in Supplementary Table 2.

### Western blot analysis

One million cells were lysed in 100 µL lysis buffer (20 mM Tris-HCl, pH 7.4, 150 mM NaCl, 1 mM EDTA, 1 mM EGTA, 10% glycerol, 1.0% NP-40, 0.1 M NaF, 1 mM DTT, 1 mM PMSF, 1 mM NaVO_4_, 2 μg/ml leupeptin and aprotinin) for 30 min on ice, and the cell extracts were treated with a Sonic Dismembrator Ultrasonic Convertor (Fisher Scientific, Pittsburgh, PA, USA)^45^. The protein concentrations of the cell extracts were quantified using the Bio-Rad Dc Protein Assay Kit (Bio-Rad Laboratories, Hercules, CA, USA). An equal amount of protein (25 μg/lane) from each cell extract was resolved on a 12% sodium dodecyl sulfate-polyacrylamide gel electrophoresis gel. The proteins were blotted onto a NOVEX NC membrane (ThermoFisher Scientific) by electrophoresis. The membranes were blocked with TBS-T (Tris-buffered saline, 0.1% Tween-20) blocking buffer (5% nonfat milk in 25 mM Tris-HCl, pH 7.4, 3 mM KCl, 140 mM NaCl, and 0.05% Tween) and subsequently probed with primary antibodies at a predetermined optimal concentration overnight at 4 °C. After extensive washing with TBS-T, the membranes were then incubated with an appropriate peroxidase-conjugated secondary antibody (Jackson ImmunoResearch Europe, Suffolk, UK) for 1 h at room temperature. After three washes with TBS-T, the blots were detected using the ECL Western Blotting Detection Reagents (EMD MILLIPORE, Newmarket, Suffolk, UK) and recorded by exposure of the blots to an X-ray film (Pierce Biotech, Rockford, IL, USA). The c-Abl band density was quantitated using the ImageJ software (http://rsbweb.nih.gov/ij) and calculated according to the β-actin band density. The primary antibodies used in the western blot analysis included HEM1 (Novus Biologicals, Littleton, CO), Abi-1 (Sigma, St. Louis, MO, USA), Abi-2 (Santa Cruz Biotech Inc., Dallas, TX, USA), WAVE2 (Cell Signaling Technology, Danvers, MA, USA), Sra-1 (Thermo Fisher Scientific, Waltham, MA, USA), c-Abl (Thermo Fisher Scientific, Waltham, MA, USA), p-Crkl (Cell Signaling technology, Danvers, MA, USA), and β-actin (Santa Cruz Biotech Inc., Dallas, TX, USA). Uncropped blots are provided in Supplementary Fig. 7.

### Lentivirus production

Lentivirus was produced after the transient infection of human embryonic kidney 293T cells with individual lentiviral vectors along with the packaging plasmids pCMV-VSV-G and psPAX2 (Addgene, Cambridge, MA, USA) using FuGEN6-HD (Roche Diagnostics, Mannheim, Germany) as the infection reagent according to Roche’s protocol^44,45^. The supernatants containing viral particles were collected 48 h after the infection and filtered through a 0.22 mm filter. The viral particles were concentrated using a PEG-itTM Virus Precipitation Solution Kit from System Biosciences (Mountain View, CA, USA) according to the manufacturer’s instructions.

### Depletion of c-Abl with shRNA

Control lentiviral pLKO.1 vectors and pLKO.1 vectors containing shRNAs for mouse c-Abl were obtained from Thermo Fisher Scientific^44,45,48^. The shRNA sequences of mouse c-Abl included clone numbers 54 (5′-AATACTCCAAA TGCCCACACG-3′), 55 (5′-AAGGTGGAT GAGTCAAAC TGC-3′), 56 (5′-TAGCTCACA CAGAAAGTG TAC-3′), 57 (5′-AACAGGTTG GTCTTTCT GTCT-3′), and 58 (5′-TACTTC TTCCAAACGCCC TCG-3′). Viral particles were produced as described above. To establish hematopoietic cells with stable c-Abl knockdown, Lin^−^ cells from E14.5 Hem-1^+/+^ embryos were maintained in Stemspan medium with TPO (10 ng/ml) and SCF (10 ng/ml) and infected twice with viral particles containing pLKO.1-c-Abl shRNA or pLKO.1-CTL shRNA under centrifugation (900 × *g*) at 32 °C for 30 min. Stably transduced cells were selected with puromycin (2 mg/ml) for 2 days. The infected cells were transplanted into 9.5 Gy lethally irradiated CD45.1 recipient mice along with 5 × 10^5^ BMCs from the recipient mice. The analysis of engraftment in the peripheral blood was performed monthly after transplantation. At 4 months post transplantation, the BMCs were used to analyze the engraftment ability. c-Abl knockdown using different shRNA clones in Lin^−^ cells was determined by western blotting as shown in Supplementary Fig. 9e.

### Transduction of constitutive c-Abl in hematopoietic cells

The pEGFP-c-Abl plasmid (generous gift from Dr. Zhi-min Yuan, Harvard T.H. Chan School of Public Health) was digested with *Bam*HI and *Xba*I. The c-Abl fragment was inserted into the *Bgl*ΙΙ and *Hpa*I sites of the pLent-GFP-vector to generate the pLent-GFP-c-Abl vector^48^. To up-regulate c-Abl expression in hematopoietic cells, FL Lin^−^ cells from E14.5 Hem-1^+/+^ and Hem-1^−/−^ embryos were maintained in Stemspan medium with TPO (10 ng/ml) and SCF (10 ng/ml) and infected twice with viral particles containing the pLent-GFP-c-Abl or pLent-GFP-vector under centrifugation (900 × *g*) at 32 °C for 30 min. Two days later, the percentages of GFP^+^ cells within the infected cells were measured with an LSRII flow cytometer and transplanted into 9.5 Gy lethally irradiated CD45.1 recipient mice along with 5 × 10^5^ BMCs from recipient mice. The engraftment ability of the GFP^+^ cells in the peripheral blood and BM was analyzed 4 months post transplantation.

### Statistical analysis

The data exhibited normal variation. No data sets were excluded from the analysis. Past experimentation was used to predetermine sample size^44,45^. The experiments were not randomized, except for the in vivo animal studies with mice as described. The investigators were not blinded to allocation during experiments and outcome assessment. The data were analyzed by analysis of variance (ANOVA). Differences among the group means were analyzed by Student–Newman–Keuls multiple comparisons test after one-way or two-way ANOVA. For experiments in which only single experimental and control groups were used, the group differences were examined by an unpaired Student’s *t* test. The frequencies of CAFC were analyzed by using Poisson statistics. The differences in the distribution of the cell cycle phases were determined by *χ*^2^ test. Survival curves were constructed using the Kaplan–Meier method and compared using the log-rank test. Differences were considered significant at *p* < 0.05. All analyses were performed with GraphPad Prism from the GraphPad software.

### Data availability

All data are available from the authors upon written request.

## Electronic supplementary material

Supplementary Information
