# Supplementary material for: The Wave2 scaffold Hem-1 is required for transition of fetal liver hematopoiesis to bone marrow
Source: Nat Commun. 2018 Jun 18;9:2377. doi: 10.1038/s41467-018-04716-5 (PMC6006146; doi:10.1038/s41467-018-04716-5)
Supplement: Supplementary file 1 — Supplementary Information [file 41467_2018_4716_MOESM1_ESM.pdf]

Supplementary Information

The Wave2 Scaffold Hem-1 is required for transition of fetal liver hematopoiesis to bone marrow

Shao et al.

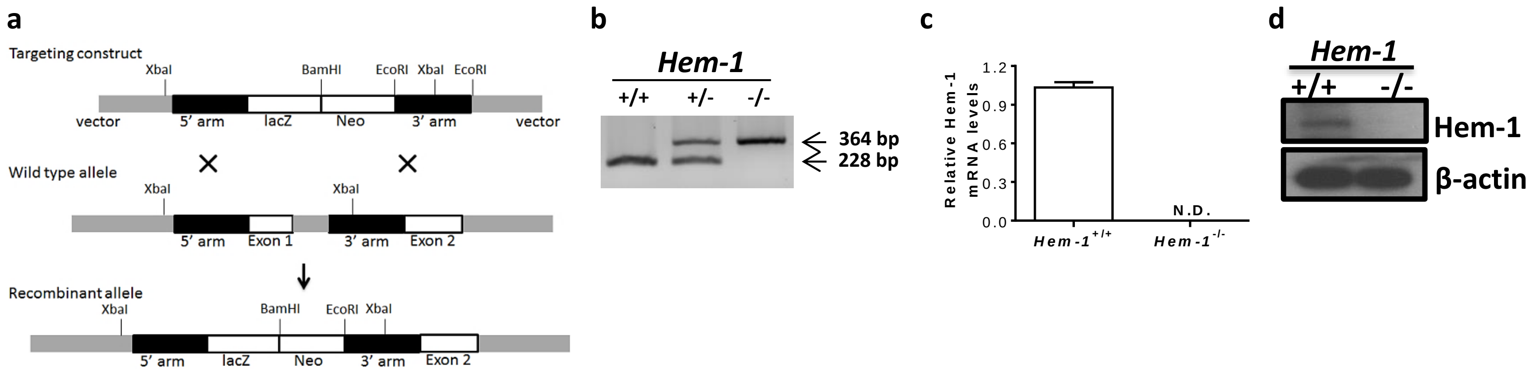

Supplementary Figure 1. Generation and Characterization of *Hem-1* knockout mice. a, Schematic diagram illustrating the targeting construct, the wild-type *Hem-1* allele, and the recombinant allele with exon 1 replaced by a lacZ/Neo cassette. **b**, Genomic PCR analysis of *Hem-1*$^{+/+}$, *Hem-1*$^{+/-}$ and *Hem-1*$^{-/-}$ mice. **c**, Expression of *Hem-1* mRNA in sorted FL LSK cells from E14.5 *Hem-1*$^{+/+}$ and *Hem-1*$^{-/-}$ embryos by qPCR (n=3 mice/group). N.D.- none detected. **d**, Representative Western blot analysis of Hem-1 expression in E14.5 *Hem-1*$^{+/+}$ and *Hem-1*$^{-/-}$ FL CD45^{+}/Lin^{-} cells. β-actin serves as the loading control.

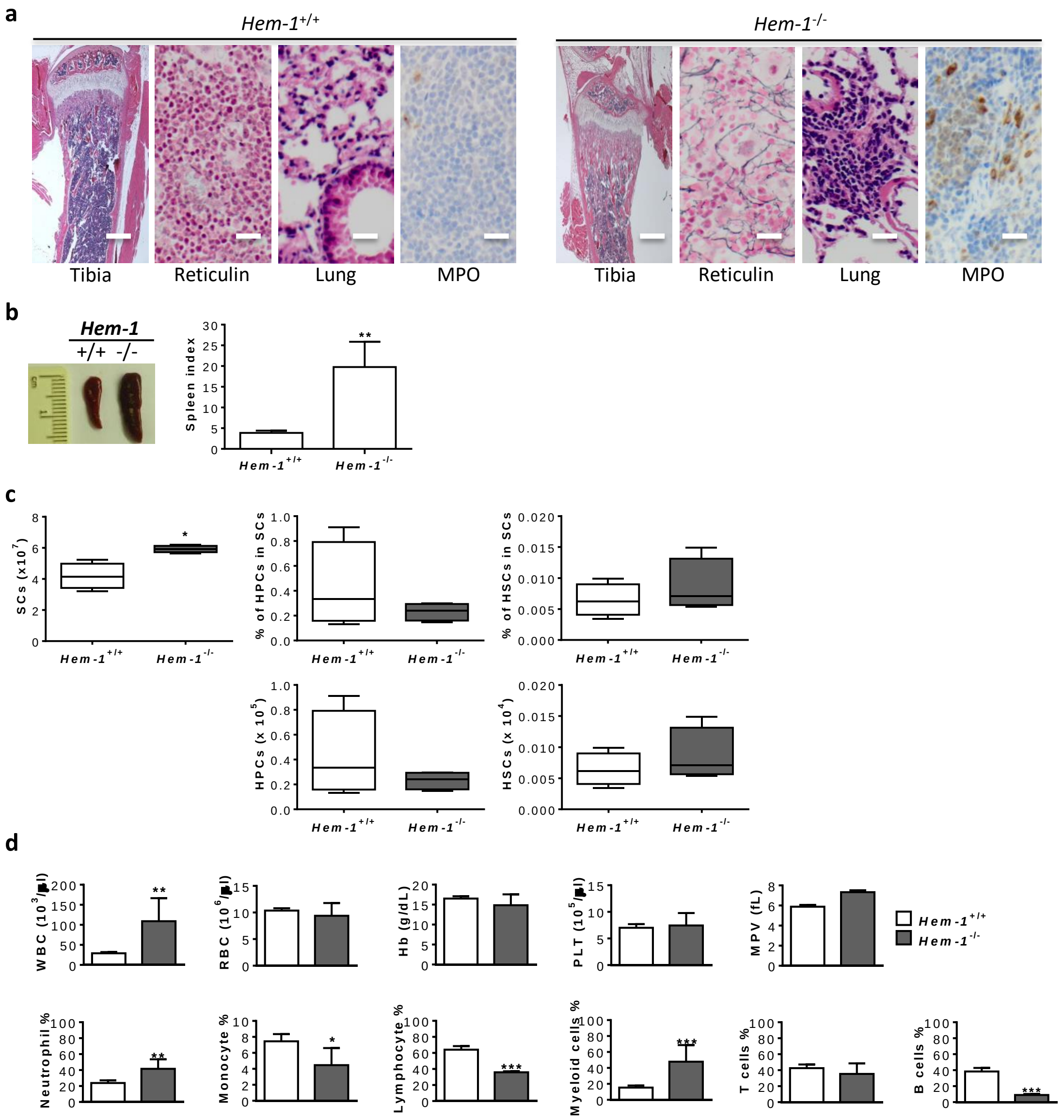

Supplementary Figure 2. *Hem-1* deletion results in leukocytosis, marrow fibrosis and extramedullary hematopoiesis. a, Representative hematoxylin and eosin staining of tibia marrow from 5-week-old mice demonstrates decreased cellularity in the *Hem-1*$^{-/-}$ mice. Representative hematoxylin and eosin staining of the lung tissue shows myeloid cell infiltration in 5-week-old *Hem-1*$^{-/-}$ mice. Representative histologic staining for reticulin in the tibia marrow and myeloperoxidase (MPO) in the spleens of 5-week-old *Hem-1*$^{+/+}$ and littermate *Hem-1*$^{-/-}$ mice is shown (n=8). Tibia scale bars approximately 500 μM, for other images, 100 μM. **b,** Anatomical appearance and volumes (spleen index) of the spleens from 5-week-old *Hem-1*$^{+/+}$ and *Hem-1*$^{-/-}$ mice (n=5, **p<0.01, Student's *t* test). **c**, Numbers of splenic nucleated cells (SCs), HPCs and

HSCs and the frequencies of HPCs and HSCs from 5-week old *Hem-1*$^{+/+}$ and littermate *Hem-1*$^{-/-}$ mice (n=5, *p<0.05, Student's *t* test). **d,** Changes in leukocytes (WBC), neutrophils, lymphocytes, monocytes, T cells, B cells, myeloid cells, erythrocytes (RBC), hemoglobin (Hb), platelets, and mean platelet volume (MPV) in the peripheral blood from 5-week-old *Hem-1*$^{+/+}$ and littermate *Hem-1*$^{-/-}$ mice (n=5, *p<0.05, **p<0.01, ***p<0.001, Student's *t* test). Error bars represent the mean ± SD.

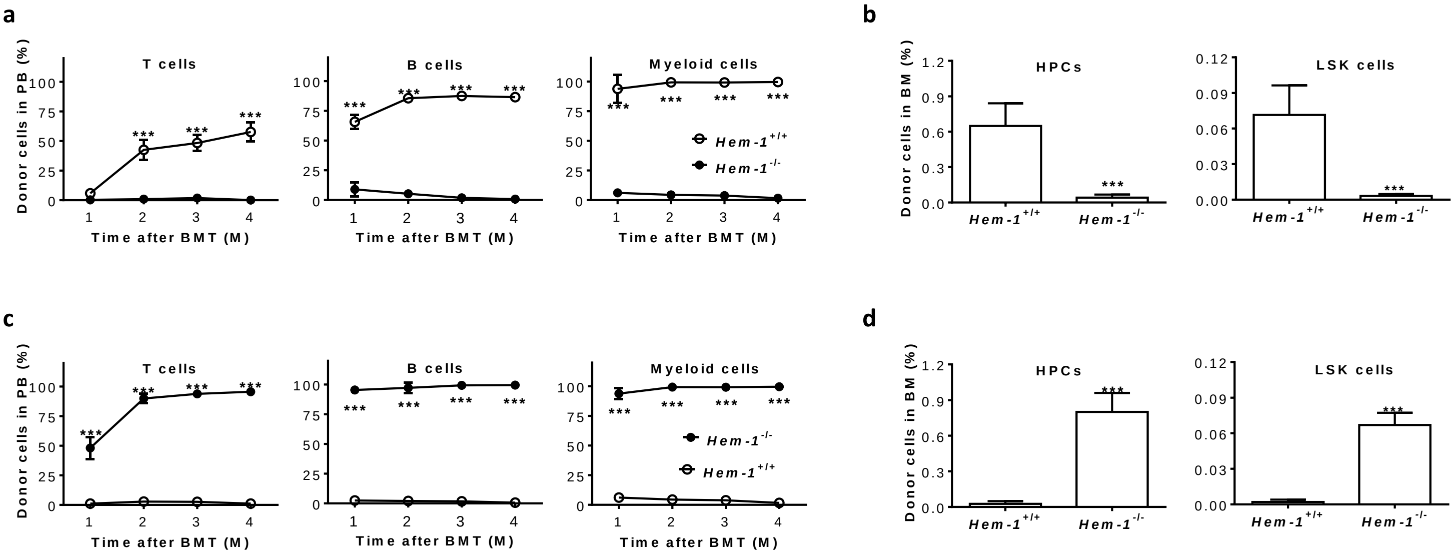

Supplementary Figure 3. *Hem-1* deletion leads to an intrinsic defect in HSCs. a, Percentages of donor-derived T cells, B cells, and myeloid cells in the peripheral blood (PB) of the lethally-irradiated normal CD45.1 recipients after receiving transplantation of FL cells from E14.5 CD45.2 *Hem-1*$^{+/+}$ and *Hem-1*$^{-/-}$ embryos in a competitive repopulation assay (CRA) as shown in **Fig. 2b**. **b**, Percentages of CD45.2 donor-derived HPCs and LSK cells in BM of the lethally irradiated normal CD45.1 recipients after receiving transplantation of FL cells from E14.5 CD45.2 *Hem-1*$^{+/+}$ and *Hem-1*$^{-/-}$ embryos in a CRA as shown in **Fig. 2b**. Data are presented in **a** and **b** as means ± SD (n=5 recipients/group). ***p<0.001, *Hem-1*$^{+/+}$ FL cells vs. *Hem-1*$^{-/-}$ FL cells, Student's *t* test. **c**, Percentages of CD45.1 donor-derived T cells, B cells, and myeloid cells in the peripheral blood (PB) of the non-ablated *Hem-1*$^{+/+}$ and *Hem-1*$^{-/-}$ CD45.2 recipients after receiving transplantation of BMCs from adult normal CD45.1 mice as shown in **Fig. 2c**. **d,** Percentages of CD45.1 donor-derived HPCs and LSK cells in BM of the non-ablated *Hem-1*$^{+/+}$ and *Hem-1*$^{-/-}$ recipients after receiving transplantation of BMCs from adult normal CD45.1 mice as shown in **Fig. 2c**. Data are presented in **c** and **d** as means ± SD (n=8 recipients/group). ***p<0.001, *Hem-1*$^{+/+}$ recipients vs. *Hem-1*$^{-/-}$ recipients, Student's *t* test.

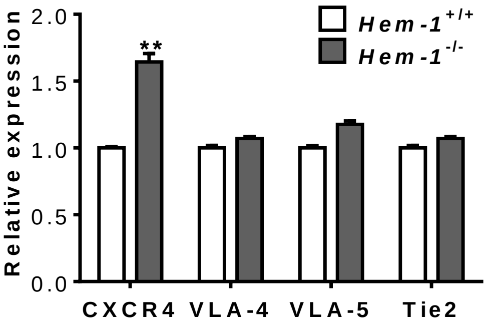

Supplementary Figure 4. *Hem-1* deletion does not reduce the expression of various adhesion molecules in FL LSK cells. The expression of CXCR4, VLA-4, VLA-5 and Tie2 mRNA in FL LSK cells from E14.5 *Hem-1*$^{+/+}$ and *Hem-1*$^{-/-}$ embryos was determined by qPCR. Data are presented as fold changes from the expression in *Hem-1*$^{+/+}$ FL LSK cells (n=3 mice/group). **p<0.01, *Hem-1*$^{+/+}$ FL LSK cells vs. *Hem-1*$^{-/-}$ FL LSK cells, Student's *t* test.

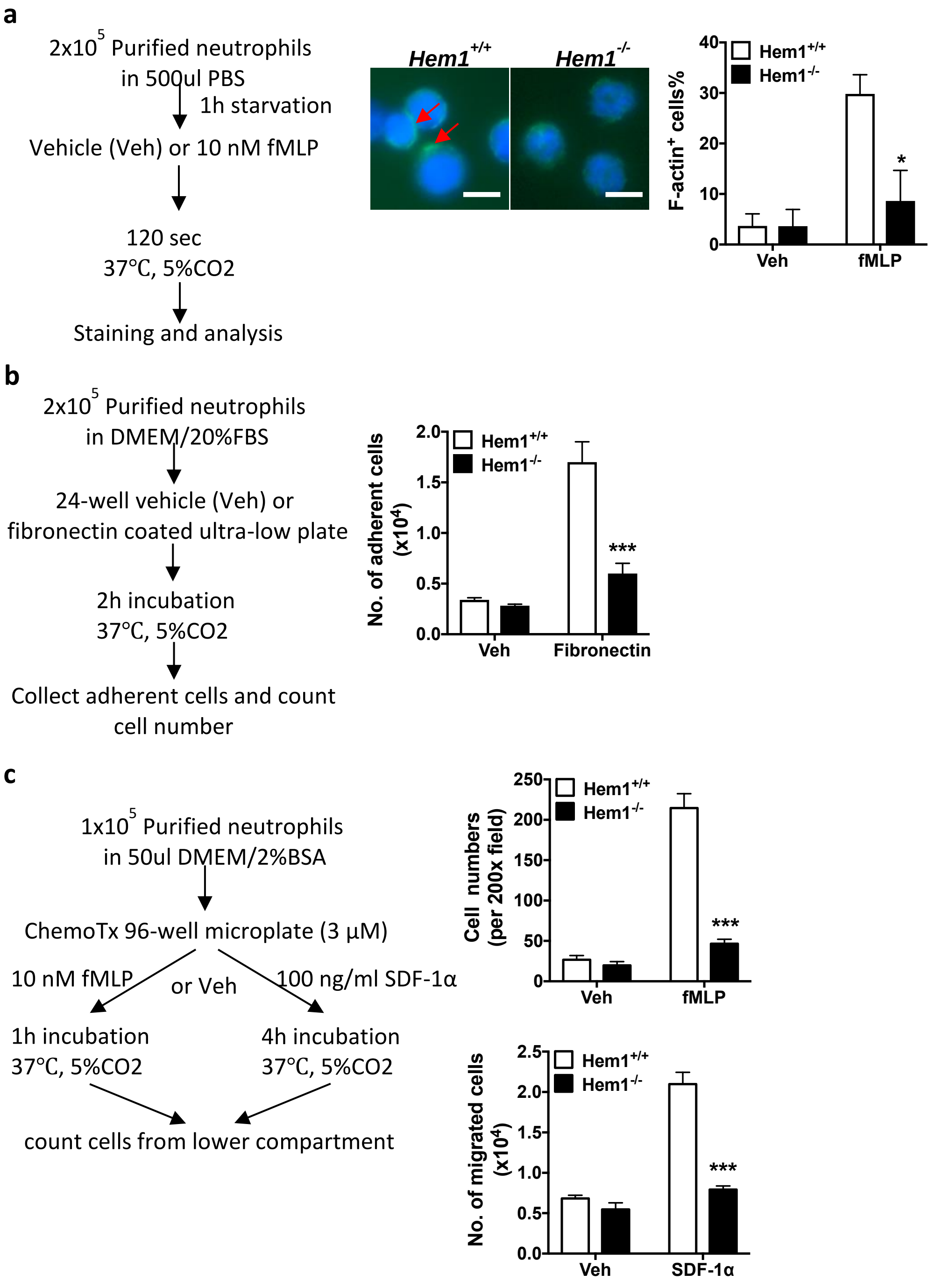

Supplementary Figure 5. Neutrophils from *Hem-1*$^{-/-}$ mice are defective in F-actin polymerization, adhesion and migration compared to the cells from *Hem-1*$^{+/+}$ mice. **a**, Neutrophils from *Hem-1*$^{-/-}$ mice are defective in fMLP-stimulated F-actin polymerization and actin capping (scale bar=10 µm). **b**, Neutrophils from *Hem-1*$^{-/-}$ mice are defective in adherence to fibronectin. **c**, Neutrophils from *Hem-1*$^{-/-}$ mice are defective in migration in response to fMLP

and SDF-1α stimulation. Data presented in the bar graphs are means ± SD (n=3/group). *$p<0.05$, ***$p<0.001$, *Hem-1*$^{+/+}$ neutrophils vs. *Hem-1*$^{-/-}$ neutrophils, two-way ANOVA.

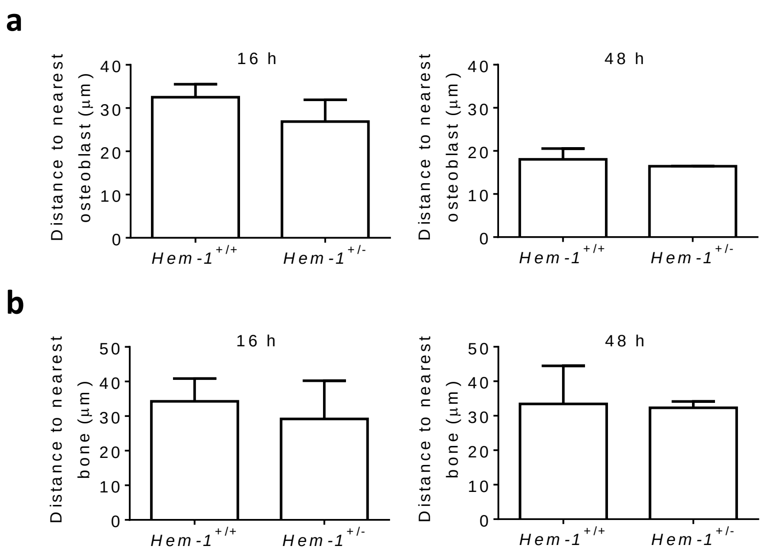

Supplementary Figure 6. *Hem-1* deletion does not impact the proximity of FL LSK cells to the osteoblastic or endosteal niches. a, *Hem-1* deletion does not impact FL LSK cell proximity to the osteoblastic niche 16 h after transplantation. In the wild type case, the FL LSK cells moved closer to the osteoblastic niche by 48 h (p=0.02, Student's *t* test). In *Hem-1*$^{-/-}$ FL LSK cells, a similar trend was present (p=0.07, Student's *t* test). **b,** There is no impact of *Hem-1* deletion on the proximity of FL LSK cells to the endosteal surface at 16 h or 48 h, and no significant change in endosteal proximity in either case between 16 h and 48 h. n=266-418 cells in 3 distinct mice.

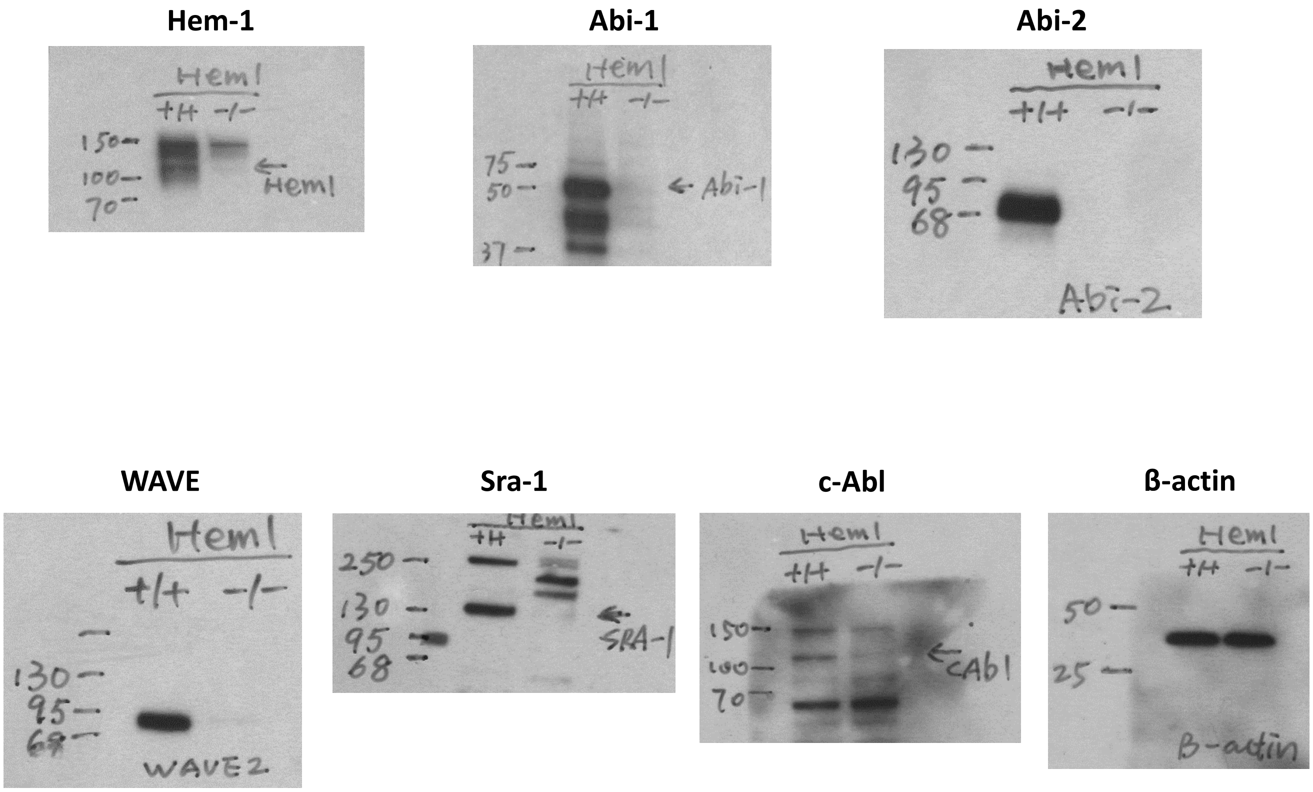

Supplementary Figure 7. Representative full size SDS-PAGE gel western analysis with MW markers for panels shown in Fig. 5a.

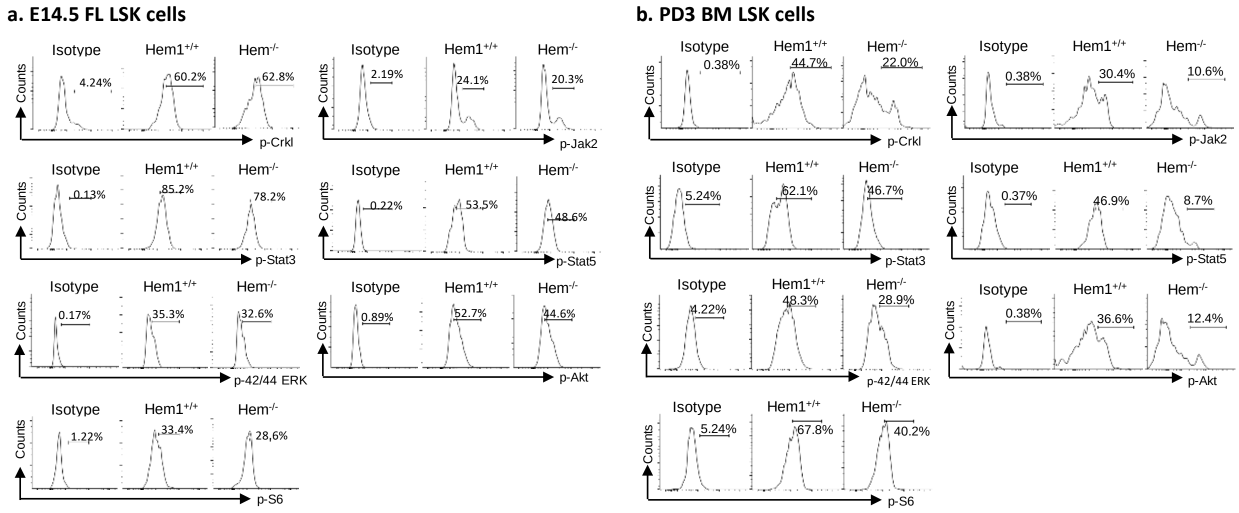

Supplementary Figure 8. PD3 *Hem-1$^{-/-}$* BM LSK cells but not E14.5 *Hem-1$^{-/-}$* FL LSK cells exhibit reduction in the phosphorylation of c-Abl downstream signaling pathway compared to littermate *Hem-1$^{+/+}$* equivalent cells. Representative phosphor flow cytometric analyses are shown.

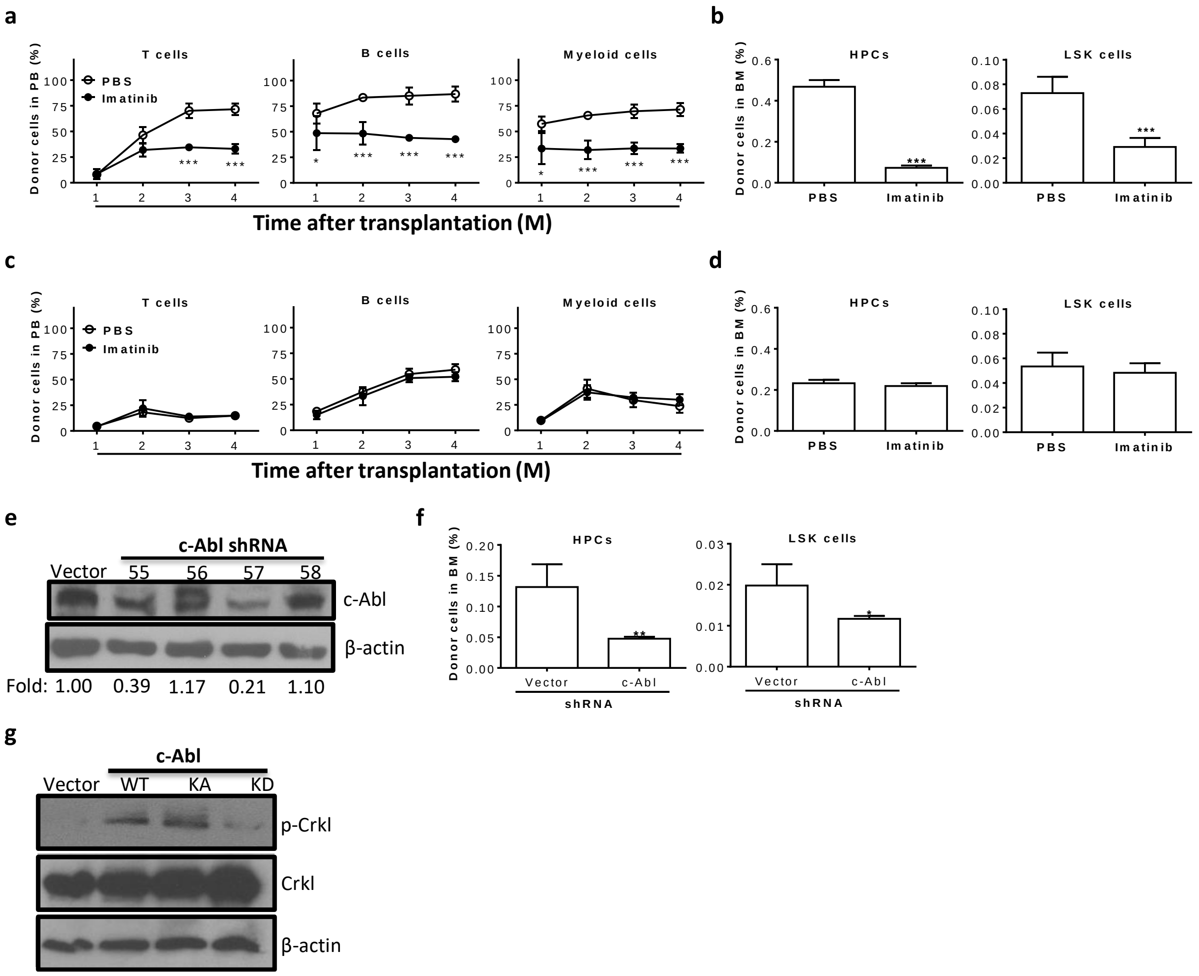

Supplementary Figure 9. Inhibition or depletion of c-Abl recapitulates the *Hem-1*$^{-/-}$ FL HSC defect in wild-type FL HSCs but not adult BM HSCs. The experimental design for **a-d** is presented in **Fig. 6e**, Percentages of donor-derived T cells, B cells, and myeloid cells in the peripheral blood (PB) of the lethally-irradiated normal CD45.1 recipients after receiving transplantation of FL Lin^{-} cells from E14.5 *Hem-1*$^{+/+}$ embryos with or without imatinib treatment. (n=5, *p<0.05, ***p<0.001, Student's *t* test). **b**, Percentages of donor-derived HPCs and LSK cells in BM of the lethally irradiated normal CD45.1 recipients after receiving transplantation of FL Lin^{-} cells from E14.5 *Hem-1*$^{+/+}$ embryos with or without imatinib treatment(n=5, ***p<0.001, Student's *t* test). **c,** Percentages of donor-derived T cells, B cells, and myeloid cells in PB of the lethally irradiated normal CD45.1 recipients after receiving transplantation of BM Lin^{-} cells from 5-week old *Hem-1*$^{+/+}$ mice with or without imatinib treatment (n=5). **d**, Percentages of donor-derived HPCs

and LSK cells in BM of the lethally irradiated normal CD45.1 recipients after receiving transplantation of BM Lin$^-$ cells from 5-week old *Hem-1*$^{+/+}$ mice with or without imatinib treatment (n=5). **e**, Representative Western blot analysis of c-Abl expression in bone marrow Lin$^-$ cells after transduction with a control vector or one of 4 different types of c-Abl shRNA (57 was chosen for HSC transduction). β-actin serves as the loading control. **f**, Percentages of donor-derived HPCs and LSK cells in BM of the lethally irradiated normal CD45.1 recipients after receiving transplantation of E14.5 *Hem-1*$^{+/+}$ FL Lin$^-$ cells transduced with control (CTL) or c-Abl shRNA in a CRA as shown in **Fig. 6h**. Data are presented in **f** as means ± SD (n=5 recipients/group). *p<0.05 and **p<0.01, c-Abl sh RNA vs. CTL shRNA, Student's *t* test. **g**, Representative Western blot analysis of phosphorylated-Crkl (p-Crkl) and Crkl expression in *Hem-1*$^{-/-}$ Lin$^-$ cells after transduction with a control vector (pLent-GFP-vector), wild-type c-Abl (WT, pLent-GFP-WT c-Abl), kinase active c-Abl (KA, pLent-GFP-KA c-Abl), or kinase dead c-Abl (KD, pLent-GFP-KD c-Abl). β-actin serves as the loading control.

Supplementary Table 1. Antibodies for flow cytometry and cell sorting

Markers	Catalogue number	Clone	Antibody isotype	Conjugate	Dilution
CD45R/B220[1]	553084	RA3-6B2	IgG_{2a}	purified	1:200
CD3e[1]	553238	145-2C11	IgG_1	purified	1:200
CD11b[1]	553308	M1/70	IgG_{2b}	purified	1:200
Gr-1[1]	553123	RB6-8C5	IgG_{2b}	purified	1:200
Ter-119[1]	553671	Ter-119	IgG_{2b}	purified	1:200
CD45R/B220[1]	553086	RA3-6B2	IgG_{2a}	biotin	1:200
CD3e[1]	553239	145-2C11	IgG_1	biotin	1:200
CD11b[1]	553309	M1/70	IgG_{2b}	biotin	1:200
Gr-1[1]	553125	RB6-8C5	IgG_{2b}	biotin	1:200
Ter-119[1]	553672	Ter-119	IgG_{2b}	biotin	1:200
CD16/CD32[1]	553140	2.4G2	IgG_{2b}	Purified	1:200
CD45.2[1]	553772	104	IgG_{2a}	FITC	1:200
CD45R/B220[1]	553092	RA3-6B2	IgG_{2a}	APC	1:200
CD45R/B220[1]	553090	RA3-6B2	IgG_{2a}	PE	1:200
CD3e[1]	565643	17A2	IgG_{2b}	APC	1:200
CD11b[1]	557397	M1/70	IgG_{2a}	PE	1:200
Gr-1[1]	553128	RB6-8C5	IgG_{2b}	PE	1:200
Streptavidin	554060	-	-	FITC	1:200
Sca-1[1]	553336	E13-161.7	IgG_{2a}	PE	1:100

Sca-1[1]	558162	D7	IgG_{2a}	PE-Cy™ 7	1:100
c-kit[1]	560185	2B8	IgG_{2b}	APC-H7	1:100
c-kit[2]	47-1171-82	2B8	IgG_{2b}	APC-eFluor® 780	1:100
CD150[2]	17-1501-81	9D1	IgG_{2a}	APC	1:100
Ki-67[2]	11-5698-82	SolA15	IgG_{2a}	FITC	1:100
CD48[3]	103418	HM481	IgG_{2a}	Pacific blue	1:200
p-Crkl(Tyr207)[4]	3181	-	IgG	Purified	1:1000
p-Jak2 (Tyr1008)[4]	8082	D4A8	IgG	Purified	1:100
p-Stat3 (Tyr705)[4]	4323	D3A7	IgG	Alexa Fluor® 488	1:100
p-Stat5 (Tyr694)[4]	3939	C71E5	IgG	Alexa Fluor® 488	1:100
p-p44/42 Erk1/2 (Thr202/Tyr204)[4]	4344	D13.14.4E	IgG	Alexa Fluor® 488	1:100
p-Akt (Ser473)[4]	4071	D9E	IgG	Alexa Fluor® 488	1:100
p-S6 (Ser235/236)[4]	4854	2F9	IgG	Alexa Fluor® 488	1:100

Footnotes: [1]BD Biosciences, San Jose, CA; [2]eBioscience, San Jose, CA; [3]Biolegend, San Diego, CA, [4]Cell Signaling technology, Danvers, MA.

Supplementary Table 2. Sequences of the primers used for qRT–PCR

Genes	Forward sequences	Reverse sequences
Bcl2	5’-ATAACGGAGGCTGGGATGCCTTTG-3’	5’-GTATGCACCCAGAGTGATGCAGGC-3’
Bcl-xl	5’-GATGGAGTAAACTGGGGTCGCATCGTG-3’	5’-AGTGTCCCAGCCGCCGTTCTCCTGGATC-3’
Mcl-1	5’-ACCAAGAAAGCTTCATCGAACCATTAGCAG-3’	5’-GGAAGAACTCCACAAACCCATCCCAGCCTC-3’
Puma	5’-GTACGAGCGGCGGAGACAAG-3’	5’-GCACCTAGTTGGGCTCCATTTCTG-3’
Bak	5’-CCTGGAACCCAACAGCATCTTGGGTC-3’	5’-GAGTTCGTAGGCATTCCCGGCTGTGG-3’
Bax	5’-TGGAGCTGCAGAGGATGATTGCTGAC-3’	5’-TTGCTAGCAAAGTAGAAGAGGGCAAC-3’
Cxcr4	5’-GCTGCACCTGTCAGTGGCTGACCTC-3’	5’-CCAGGATGAGAACGCTGCTGTAGAG-3’
Vla-4	5’-ACAATGAGCCATTACTATGATTATC-3’	5’-CAAATTCTTCAATTCCTTGTAAACAG-3’
Vla-5	5’-TTGTCAGACACCCAGGGAACTTCTC-3’	5’-CGAAGGCCACCCCAGAGACTGGTGC-3’
Tie2	5’-GGGTTGCAGTGCAATGAAGCATGCC-3’	5’-TCATCCTTGGCCTGCCTTCTTTCTC-3’
HPRT	5’-AGCAGTACAGCCCCAAAATGGTTA-3’	5’-TCAAGGGCATATCCAACAACAAAC-3
